# High Glucose in Diabetic Hyperglycemia Perturbs Lymphocyte SERCA-Regulated Ca^2+^ Stores with Accompanying ER Stress and Signaling Dysfunction

**DOI:** 10.3390/biom15070987

**Published:** 2025-07-11

**Authors:** Md Nasim Uddin, James L. Graham, Peter J. Havel, Roshanak Rahimian, David W. Thomas

**Affiliations:** 1 Department of Pharmaceutical Sciences, Thomas J. Long School of Pharmacy, University of the Pacific, Stockton, CA 95211, USA; m_uddin1@u.pacific.edu (M.N.U.); rrahimian@pacific.edu (R.R.); 2 Department of Molecular Biosciences, School of Veterinary Medicine, University of California, Davis, CA 95616, USA; jlgraham@ucdavis.edu (J.L.G.); pjhavel@ucdavis.edu (P.J.H.); 3 Department of Nutrition, University of California, Davis, CA 95616, USA; 4 Department of Cellular and Physiological Sciences, University of British Colombia, Vancouver, BC V6T 1Z3, Canada

**Keywords:** type 2 diabetes (T2D), hyperglycemia, T cell signaling, endoplasmic reticulum (ER) stress, Ca^2+^ signaling, ER Ca^2+^ release, Ca^2+^ stores, T cell dysfunction

## Abstract

It is well recognized that patients with type 2 diabetes mellitus (T2DM) exhibit significant impairment of immune function resulting in a higher frequency of infections. We hypothesize in this study that a likely contributor to immune dysfunction in T2DM is alteration of T lymphocyte signaling functions induced by chronic hyperglycemia. In this study we have utilized the established UC Davis Type 2 Diabetes Mellitus (UCD-T2DM) rat model of human T2DM to investigate whether progressive hyperglycemia diminishes T cell receptor (TCR)-releasable endoplasmic reticulum (ER) Ca^2+^ stores, an essential early antigen-stimulated signal driving T cell activation. Furthermore, results from this study demonstrate that chronic hyperglycemia markedly alters the expression profile of the sarco/endoplasmic reticulum Ca^2+^-ATPase (SERCA) Ca^2+^ ion pumps, which are the major enzymatic ion transporters maintaining replenished TCR-sensitive Ca^2+^ pools. We conducted companion experiments using Jurkat T lymphocytes exposed to high glucose which allowed finer resolution of early disruptions to ER Ca^2+^ store integrity and greater clarity on SERCA isoform-specific roles in diabetes-induced Ca^2+^ signal dysregulation. In summary, these experiments suggest that hyperglycemia in T2DM drives an ER stress state manifesting in reduced expression of the SERCA pumps, erosion of ER Ca^2+^ stores and culminating in T cell and immune dysfunction.

## 1. Introduction

Chronically elevated blood glucose levels as a primary driver of severe vascular dysfunction in type 2 diabetes (T2DM) are also suspected to be a major etiological factor underlying the pronounced immune system dysfunction associated with the disease [1,2]. Indeed, it is well known that patients with T2DM, along with multiple other pathological conditions, also suffer from a higher incidence of infections compared to unaffected individuals, suggesting a broadly compromised immune function [1]. Given the central role of the T lymphocyte in coordinating and regulating multiple key elements of the immune response, we propose that a cogent hypothesis for immune function impairment is the likely deleterious effect of chronic hyperglycemia and energy disruptions on essential T cell signaling functions.

The T cell initiates the complex sequence of the adaptive immune response following T cell receptor (TCR)-induced transmission of foreign antigen recognition into a cellular activation and proliferation regimen committed to eradication of infectious threats to the host [3]. Moreover, one of the key signaling pathways which the TCR recruits to achieve T cell activation is the production of inositol 1,4,5-trisphosphate (IP3) with the accompanying release of Ca^2+^ from the endoplasmic reticulum (ER) [4,5,6]. The release of Ca^2+^ from the internal ER Ca^2+^ store effectively produces an ER depletion signal that couples to activation of the plasma membrane-localized Orai1 Ca^2+^ channel resulting in sustained Ca^2+^ influx [7,8]. Indeed, full T cell activation and differentiation fails in the absence of this two-phase Ca^2+^ release/influx TCR-activated pathway and thereby results in a pronounced immunodeficient state [9,10]. Working in concert with, and rhythmically integrated within, this TCR-induced Ca^2+^ signal cycle are the major Sarco/Endoplasmic Reticulum Ca^2+^-ATPase (SERCA) ion-transporting pumps [11]. SERCA pumps consume cellular ATP driving their solute-transporting enzymatic cycle of pumping Ca^2+^ ions, against energetically unfavorable gradients, back into the ER, and critically maintaining sequestered ER Ca^2+^ at high levels [12,13]. Thus, the SERCA pumps regulate the critical TCR-activated Ca^2+^ signal on two levels: firstly by ensuring TCR-releasable ER Ca^2+^ levels are sufficient and, secondly, by influencing the degree of ER Ca^2+^ depletion, SERCA pump activity determines the scale of Orai1-mediated Ca^2+^ influx which, in turn, is linked to the fidelity and proficiency of T-cell activation [4,5].

Notably, several studies have reported findings that demonstrate both impaired SERCA function and reduced SERCA expression levels in T2DM in tissues of the cardiovascular system [14,15,16]. However, investigation of immune cell SERCA defects in the presence of chronic hyperglycemia have not been adequately pursued. We thus propose the hypothesis that SERCA dysfunction, due to sustained high blood glucose with consequent cellular energy disruptions, results in T cell functional deficits, and likely serves as a major contributing factor to T2D immunodeficiency. We have used the UC Davis Type 2 Diabetes Mellitus (UCD-T2DM) rat model of T2DM to isolate lymphocytes and examine the effects of chronic hyperglycemia on SERCA control of ER Ca^2+^ signaling functions. The UCD-T2DM model is a well-established animal model that more closely mimics the development and progression of T2DM in humans, with a characteristic time-dependent onset of hyperglycemia along with other pathologic sequelae shared with the human form of the disease [17,18,19]. Specifically, the animals exhibit key features of the human form of T2DM such as, polygenic adult-onset obesity, insulin resistance, intact leptin signaling and pancreatic β-cell decompensation [17,18]. Thus, based on these similarities, the UCD-T2DM model can provide us with much needed data on the pathophysiological mechanisms underlying the development of human T2DM in human patients.

Here, we report progressive ER Ca^2+^ store degradation in lymphocytes obtained from UCD-T2DM rats exposed to chronic hyperglycemia, revealing impaired SERCA pump expression and manifesting in severely depleted ER Ca^2+^ stores and induction of ER stress mediators. We also performed complementary studies using high-glucose incubation experiments in Jurkat T lymphocytes to enable more detailed investigation of early perturbations induced by high-glucose stress. Moreover, use of clonal homogeneous Jurkat T cells enabled the performance of experiments previously conducted, which illuminated the distinct roles played by T cell SERCA pump isoforms in managing high-glucose ER stress and helped clarify potentially productive pharmacological strategies for preserving immune cell function in the diabetic condition [20,21].

## 2. Materials and Methods

### 2.1. Materials

Fura 2/AM (Fura-2 acetoxymethyl ester), Fluo-3 pentapotassium salt, pluronic acid, RPMI-1640, fetal bovine serum (FBS), streptomycin, penicillin, GlutaMAX, MEM vitamin solution, MEM amino acid solution, sodium pyruvate and HBSS (Hanks Balanced Salt Solution) were obtained from Thermo Fisher Scientific (Waltham, MA, USA). Thapsigargin, oligomycin and tissue culture flasks were obtained from Santa Cruz Biotechnology, Inc. (Dallas, TX, USA). D-myo-Inositol 1,4,5-trisphosphate potassium salt (IP3), phytohemagglutinin (PHA), 2,5-di-(tert butyl)-1,4-benzohydroquinone (tBHQ), creatine phosphokinase (CPK), phosphocreatine disodium salt hydrate, adenosine 5′-triphosphate disodium salt hydrate (ATP), DTT, β mercaptoethanol and saponin were obtained from Sigma-Aldrich (St. Louis, MO, USA). Sterile cell strainers (100 μm), 50 mL syringe tubes and 60 mm cell culture dishes were obtained from Fisher Scientific. CDN1163 and cyclopiazonic acid were from Bio-Techne (Minneapolis, MN, USA).

### 2.2. Cell Culture

Jurkat cells (Clone E6–1, ATCC TIB-152) were maintained in RPMI-1640 medium supplemented with 10% fetal bovine serum, 2 mM L-glutamine, penicillin (100 IU/mL) and streptomycin (100 μg/mL) at 37 °C in a humidified atmosphere (95% air, 5% CO_2_). Jurkat cells were cultured under various glucose concentration conditions including maintenance level glucose (5.5 mM, denoted as normal), moderately elevated glucose (15 mM, denoted as intermediate high) and aggressively elevated glucose (25 mM, denoted as high) to model hyperglycemic diabetic conditions. Mannitol was used as an osmotic control for the high glucose experimental condition. Cell cultures were maintained at a cell concentration between 1 × 10^5^ and 1 × 10^6^ viable cells/mL in either 25 cm^2^ (T25) or 75 cm^2^ (T75) tissue culture flasks, and fresh medium was added every 2 to 3 days depending on cell density.

### 2.3. Isolation and Culture of Primary Lymphocytes from UCD-T2DM Rats

The use of animals for these experiments was conducted in accordance with protocols approved by the Institutional Animal Care and Use Committees at the University of the Pacific. UCD-T2DM rats used in this study were generated by crossing obese insulin-resistant Sprague Dawley (SD) rats with Zucker diabetic fatty (ZDF) lean rats, producing a population with clinical features closely resembling the pathophysiology of T2DM in humans [17]. Given the similarity to human T2DM, the UCD-T2DM rat model has gained popularity and increasing usage with numerous studies employing the model for investigations into the molecular/cellular pathogenesis of diabetes [18]. For the purpose of this study, we selected 22–24-week-old rats that had not yet developed diabetes (pre-diabetic group) and 22–24-week-old rats that had developed diabetes (diabetic group), maintaining these animals following selection for experimental investigation at the 3- and 6-month intervals. The rats were bred in the animal facility of the Department of Nutrition at UC Davis followed by transfer and maintenance at the University of the Pacific vivarium in standard shoebox cages on a 12 h light:dark cycle with water and food ad libitum. After transferal and acclimation for 1 week at the animal facility, progression of diabetic phases was determined by measuring blood glucose levels in 12 h fasted rats using a standard glucose test meter (OneTouch, LifeScan Milpitas CA USA) and measuring HbA1c levels using the A1cNow+ test kit (PTS Diagnostics Sunnyvale, CA, USA), according to the manufacturer’s instructions. On the day of sacrifice, spleens were isolated from UCD-T2DM rats at the pre-diabetic stage (PD; *n* = 6), as well as after 3 months (D3M: *n* = 6) and 6 months (D6M, *n* = 3) in the diabetic stage. Briefly, rats were anesthetized, and spleens were aseptically removed and placed into 60 mm cell culture dishes containing ice-cold HBSS and minced into small pieces with a scissor. Tissue fragments were dissected and passed through a 100 μm cell strainer using a 10 mL syringe plunger and ice-cold HBSS into a 50 mL conical tube, then centrifuged (200× *g* at 4 °C) for 10 min. The supernatant was discarded, and the pellet was resuspended in 5 mL of a red blood cell (RBC) lysis buffer containing 155 mM NH_4_Cl (9 parts) in 130 mM Tris base pH 7.65 (1 part) and incubated at 37 °C for 5 min. RBC lysis was halted by the addition of 10 mL of ice-cold complete cell culture medium supplemented with 10% FBS, and cells were then centrifuged (200× *g* at 4 °C) for 10 min. The supernatant was discarded, and the pellet was resuspended in 10 mL of complete cell culture medium (RPMI 1640 medium supplemented with 10% FBS, 2mM L-glutamine, 2% GlutaMAX, 1% MEM vitamin solution, 1% MEM amino acid solution, 1% sodium pyruvate, 0.01% β mercaptoethanol). Cells were counted using a hemocytometer with Trypan Blue vital dye exclusion and maintained in a humidified atmosphere (37 °C, 95% air, 5% CO_2_).

### 2.4. Activation of Lymphocytes

A portion (10^6^ cells/mL) of the isolated lymphocytes were incubated overnight without stimulation (denoted as resting lymphocytes) and used to measure intracellular Ca^2+^ responses as well as harvested for material for Western blot analysis within 24 h after isolation. Another portion (10^6^ cells/mL) of lymphocytes was activated (referred to as activated lymphocytes) on the day of isolation with 10 μg/mL phytohemagglutinin (PHA), which cross-links T cell receptors. Activated lymphocytes were used to measure intracellular Ca^2+^ and for preparation of samples for Western blot analysis within 72 h of activation.

### 2.5. Cell Calcium Assays

Cells (approximately 1 × 10^6^ cells/mL) were washed in Ca^2+^-containing (1.8 mM) HBSS and loaded with 1.5 μM Fura-2/AM in 20% (*w*/*v*) Pluronic F-127 and incubated for one hour at 37 °C. After loading, the cells were washed twice with HBSS and incubated at 37 °C for an additional 30 min to allow for de-esterification of the dye. Cells loaded with Fura 2/AM were kept in the dark at room temperature throughout the experiments. Changes in cytosolic Ca^2+^ were measured in cell population experiments using a fluorescence spectrophotometer equipped with a thermostatically controlled sample compartment (PTI, Lawrenceville, NJ, USA), permitting continuous stirring of samples in the cuvette. All measurements were carried out at room temperature (25 °C). To achieve Ca^2+^-free conditions, EGTA (2 mM) was added to chelate extracellular Ca^2+^ just before the addition of Ca^2+^ mobilizing agonists (1–2 min). Ca^2+^ changes in Jurkat cells and UCD-T2DM rat splenocytes loaded with Fura 2/AM were measured via rapid alternation of the excitation monochromator between 340 and 380 nm, with fluorescence emission measured at 510 nm using a ratiometric spectrofluorometer (PTI, Lawrenceville, NJ, USA). Cytosolic Ca^2+^ responses are presented as the changes in the fluorescence ratio values measured at 340/380 nm for Fura-2 or as non-ratiometric Fluo-3 fluorescence changes for the excitation/emission (503/530 nm) wavelength pair. The data are reported as either peak amplitude changes in fluorescence values or as initial rates of fluorescence changes and presented as the means ± S.E.M.

### 2.6. Permeabilized Cell Assays

For preparation of permeabilized cells, 4 × 10^7^ cells were washed twice and resuspended in 2 mL of an intracellular-like medium (ICM: 110 mM KCl, 10 mM NaCl, 2 mM MgCl2, 20 mM HEPES, 5 mM KH_2_PO_4_, pH 7.5) in the presence of 1 mM DTT. Saponin (20 μg/mL) was added, and the cell suspension was incubated for 5 min at 37 °C to complete permeabilization. An ATP-regenerating system consisting of creatine kinase (40 units/mL) and phosphocreatine (20 mM) was added. Oligomycin (10 μg/mL) was also included to inhibit the mitochondrial ATPase. Following cell permeabilization, Fluo-3 (0.5 μM) was added to the cuvette. Subsequent addition of ATP to a final concentration of 1 mM resulted in a decrease in the fluorescence, indicating Ca^2+^ uptake by the intracellular stores. After baseline stabilization, reagents and Ca^2+^ agonists were added and Fluo-3 fluorescence changes were recorded. Ca^2+^ release from intracellular stores was measured from cells suspended in cuvettes using a fluorescence spectrophotometer equipped with a thermostatically controlled sample compartment maintained at 37 °C with continuous stirring. Fluorescence changes with Fluo-3 in permeabilized cell suspensions were measured with excitation wavelength settings of 503 nm and 530 nm for the emission wavelength, and Ca^2+^ uptake rates were determined by computing linear initial rates of Fluo-3 fluorescence changes after the provision of ATP to the cell suspension.

### 2.7. Western Blot Analysis

To evaluate SERCA and ER stress protein expression levels, Western blotting was performed using standard methodologies. In brief, cells were collected from culture flasks and pelleted via centrifugation. After washing with ice-cold phosphate-buffered saline, cell pellets were lysed with ice-cold RIPA buffer supplemented with 1× Halt protease and phosphatase inhibitor (Thermo Fisher Scientific, Waltham, MA, USA) for 20 min on ice. Whole-cell lysates were clarified by centrifugation (15,000× *g* for 15 min at 4 °C), and total protein concentration was determined by the Pierce BCA assay (Thermo Fisher Scientific, Waltham, MA, USA) according to manufacturer protocols. Samples were analyzed using sodium dodecyl sulfate-polyacrylamide gel (SDS-PAGE) electrophoresis and transferred to low-fluorescent PVDF membranes (Bio-Rad, Hercules, CA, USA) by wet transfer. Membranes were blocked for 1 h at room temperature with Intercept Blocking Buffer (LI-COR, Lincoln, NE, USA) and incubated overnight at 4 °C with primary antibodies: anti-SERCA 2, anti-Stim1, anti-ATF6, anti-β Actin (Santa Cruz Biotechnology, Dallas, TX, USA), anti-SERCA 3 (Thermo Fisher Scientific, Waltham, MA, USA), and anti-Phospho-eIF2α (Cell Signaling Technology, Danvers, MA, USA). After treatment with primary antibodies, the membranes were washed four times for 5 min with washing buffer (TBS-T) and incubated with the IRDye 800CW secondary antibodies (LI-COR, Lincoln, NE, USA) for 1 h at room temperature. The bands of proteins were detected by the LI-COR Odyssey M imaging system using Image Studio software Version 6.0 (Lincoln, NE, USA). β-Actin was used as loading control for all Western blots. Where necessary, blots were washed once for 5 min with TBS-T, then stripped for 25 min at room temperature using NewBlot IR Stripping Buffer (LI-COR) with gentle agitation. After stripping, the blots were re-blocked, incubated with primary antibody, washed, incubated with secondary antibody, washed again and subsequently developed and imaged following the same procedure as before.

### 2.8. RNA Extraction, Purification, Reverse Transcription and RT-qPCR Analysis

Extraction and purification of RNA from Jurkat T cells was performed using RNeasy Protect Mini Kit (Qiagen, Valencia, CA, USA) following the manufacturer’s protocol. Total RNA was measured using a Nanodrop Lite Spectrophotometer (Thermo Fisher Scientific), and 1 μg/sample of total RNA was used for reverse transcription with elimination of genomic DNA using QuantiTect Reverse Transcription Kit (Qiagen) according to the manufacturer’s instructions. Real-time quantitative PCR was performed using TaqMan™ Fast Advanced Master Mix and TaqMan^®^ Assay primer/probe (fluorogenic probe-primer combinations specific for the target gene; Assay ID: Hs00544877_m1 ATP2A2, Hs01024563_m1 ATP2A3, Hs02758991_g1 GAPDH, Hs00231936_m1 XBP1, Hs00358796_g1 DDIT3/CHOP, Hs00607129_gH HSPA5/GRP78, Hs01115905_m1 PDIA4/ERP72) (Thermo Fisher Scientific). The cycling conditions comprised 2 min Uracil-N-glycosylase activation at 50 °C, 20 s polymerase activation at 95 °C, 40 cycles of denaturation at 95 °C for 3 s and annealing/extension at 60 °C for 30 s. Reactions were run on the Bio-Rad CFX96™ Real-Time PCR System (Bio-Rad, Hercules, CA, USA) according to the manufacturer’s protocol. The relative expression levels of target genes were determined by the 2^–ΔΔCq^ method of quantifying mRNA levels using GAPDH as the reference housekeeping gene.

### 2.9. Cell Viability Assay

The viability of Jurkat lymphocytes was measured using the CellTiter 96 AQ_ueous_ Non-Radioactive Cell Proliferation Assay (MTS) system (Promega) according to the manufacturer’s instructions. Cells were seeded in a multi-well plate and cultured under control (5.5 mM glucose) and high glucose (25 mM glucose) conditions, and viability was assessed every 24 h over a 4-day period. In separate experiments, cells treated with various pharmacological agents were plated into each well of a 96-well plate and at the conclusion of the experiment 20 μL of MTS reagent was added to the well. After 3.5 h of incubation at 37 °C in a humidified, 5% CO_2_ atmosphere, absorbance at 490 nm was measured using a microplate reader (Spectramax ID3, Molecular Devices, USA) to determine the viability of the cells, which was expressed as a percentage of the control group. Three replicate wells per experimental condition were used to obtain measures of cell proliferation.

### 2.10. Statistical Analysis:

For Ca^2+^ measurements in cell populations statistical significance was determined using two-tailed, unpaired Student’s *t*-test, and differences were considered significant if *p* < 0.05. Comparisons between more than two groups were carried out by one-way ANOVA and two-way ANOVA employing Tukey’s, Dunnett’s and Sidak’s multiple comparisons test where applicable, with *p* < 0.05 determining significance. Statistical analyses were performed using GraphPad prism 10.3 and later versions (GraphPad Software Inc., San Diego, CA, USA).

## 3. Results

### 3.1. Lymphocytes Exposed to Chronic Hyperglycemia Exhibit Altered Ca^2+^ Signaling, Reduced SERCA Expression and Depleted ER Ca^2+^ Stores with ER Stress Activation

**Figure 1:**
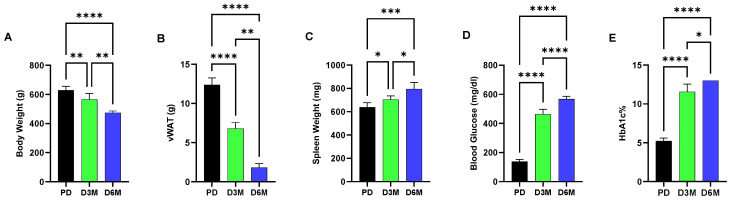
Assessment of metabolic parameters of UCD-T2DM rats over the prediabetic (PD) to three-month (D3M) and six-month (D6M) diabetic interval. (**A**). Body weight (**B**). Visceral white adipose tissue (vWAT) weight (**C**). Spleen weight (**D**). Blood glucose levels (**E**). Hemoglobin A1C (HbA1c%) levels. Values are presented as mean ± SEM. Each bar represents values obtained from *n* = 3–6 animals per group. Asterisks denote statistical significance between two groups with * *p* < 0.05, ** *p* < 0.005, *** *p* < 0.001, **** *p* < 0.0001, analyzed by one-way ANOVA followed by Tukey’s post hoc test.

Figure 1 shows changes over a six-month interval in UCD-T2DM rats in blood glucose, hemoglobin A1c (HbA1c), body and adipose (visceral white adipose tissue, vWAT) weight and spleen weight. These metabolic parameters show the progression of diabetic pathogenesis in UCD-T2DM rats from the prediabetic (PD) state to elevated blood glucose and HbA1c levels at both the 3-month (D3M) and 6-month (D6M) time points. Accompanying the high blood glucose condition, we observed a significant progressive loss in body weight (10% reduction in D3M and 23% reduction in D6M) and adipose tissue, while in contrast we observed a modest increase in spleen weight (7% increment in D3M and 20% increment in D6M, Figure 1). Increased spleen weight is likely due to increased inflammatory conditions associated with diabetic progression.

**Figure 2:**
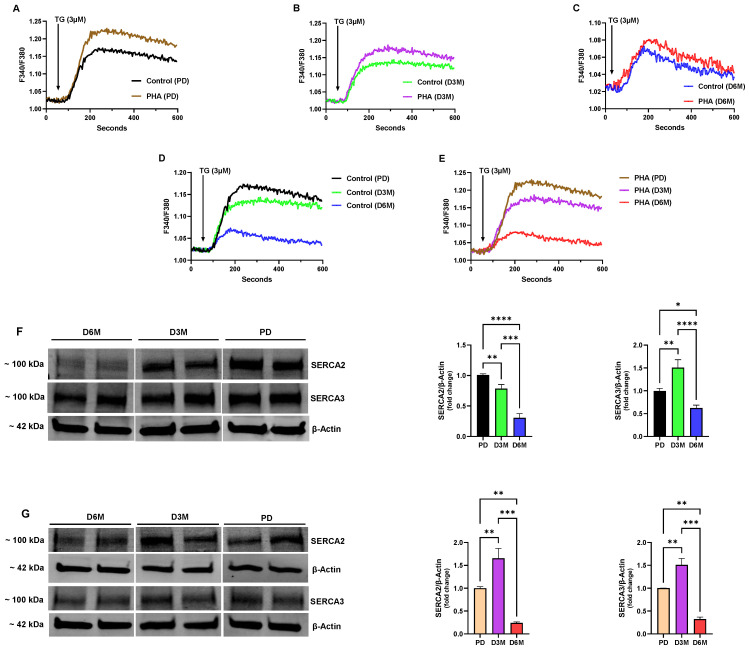
Progressive diminishment in ER Ca^2+^ release responses and altered SERCA expression levels in unstimulated and PHA-stimulated UCD-T2DM rat lymphocytes subjected to chronic hyperglycemia. For A-E UCD-T2DM rat spleen lymphocytes were loaded with Fura-2 and suspended in Ca^2+^-free media (balanced salt solution plus 2 mM EGTA). (**A**) Rat lymphocytes from the pre-diabetic (PD) stage were unstimulated or stimulated with PHA (10 µg/mL, 72 h, red trace) and Ca^2+^ release was induced by application of TG (3 μM). (**B**) Same experiment as in (**A**) but lymphocytes were isolated from the three-month diabetic (D3M) stage and treated with TG (3 μM). (**C**) Same experiment as in (**B**) but lymphocytes were isolated, followed by either no treatment or PHA stimulation (10 µg/mL, 72 h) and challenged with TG (3 μM) at the six-month (D6M) diabetic stage. (**D**) TG (3 μM)-induced Ca^2+^ release responses in rat lymphocytes isolated from the PD, D3M and D6M stages of hyperglycemia. (**E**) Same experiment as described in (**D**) but using rat lymphocytes stimulated with PHA treatment (10 µg/mL, 72 h). (**F**) Representative Western blot image of SERCA 2b and SERCA 3 Ca^2+^-ATPase isoforms in unstimulated UCD-T2DM rat lymphocytes. Figure shows approximate molecular weight of SERCA proteins with β-actin protein bands as control. Also shown are densitometry bar plots derived from band density quantification and normalized to β-actin protein levels for SERCA 2b (*green bar*) and SERCA 3 (*blue bar*). Each bar represents average ± SEM of *n* = 3–6 animals. (**G**) Representative Western blot image of SERCA 2b and SERCA 3 Ca^2+^-ATPase isoforms in PHA-stimulated UCD-T2DM rat lymphocytes. Figure shows approximate molecular weight of SERCA proteins with β-actin protein bands as control. Also shown are densitometry bar plots derived from band density quantification and normalized to β-actin protein levels for SERCA 2b (*purple bar*) and SERCA 3 (*red bar*). Each bar represents average ± SEM of *n* = 3–6 animals. Fluorescence traces of Ca^2+^ experiments shown are representative of three to five separate experiments with significant differences assessed via use of Student’s t test. One-way ANOVA followed by Tukey’s multiple comparison tests were used to analyze Western blot data for SERCA expression levels with *n* = 3–6. Asterisks denote statistical significance with * *p* < 0.05, ** *p* < 0.005, *** *p* < 0.001 and **** *p* < 0.0001.

In this study we were prompted to examine the hypothesis that lymphocytes isolated from animals experiencing chronic hyperglycemia would demonstrate, due to profoundly disrupted metabolism, altered Ca^2+^ signaling parameters and ER homeostasis defects that likely underlie immunes system dysfunction in the diabetic state. Figure 2 shows the changes in ER Ca^2+^ stores from the pre-diabetic (PD) state to animals experiencing chronic high blood glucose for 3- and 6-month intervals (D3M and D6M). We used thapsigargin (TG, 3μM) to release internal Ca^2+^ stores, employing a TG concentration that is expected to effectively block all SERCA pump isoforms and release the composite SERCA-regulated Ca^2+^ pools [22,23]. For these experiments we used rat spleen lymphocytes in both the resting and activated (PHA treated) state. PHA is a well-known T cell mitogen via its action to crosslink and activate the T cell receptor (TCR), and thus enabled our approach to preferentially measure Ca^2+^ responses in the activated T cell population [24,25]. We observed a significantly greater amplitude response in the PD UCD-T2DM lymphocytes stimulated with PHA treatment (10 µg/mL, 72 h) compared to untreated lymphocytes (ΔF 0.208 ± 0.006 PHA-treated vs. 0.152 ± 0.009 Control *p* < 0.05, *n* = 6–8, Figure 2A), suggesting that activation of the T cells produced an expanded Ca^2+^ store capacity in the global SERCA-regulated Ca^2+^ pools. At the three-month stage (D3M), in rats experiencing significantly higher blood glucose levels (Figure 1C), we found that the TG-induced Ca^2+^ release response was substantially diminished (PD PHA-treated: ΔF 0.208 ± 0.006 vs. D3M PHA-treated: ΔF 0.160 ± 0.004, *p* < 0.05, *n* = 6–8, Figure 2A vs. Figure 2B); and, while the PHA-stimulated lymphocytes still produced a greater amplitude response than unstimulated lymphocytes, the augmentation of the Ca^2+^ release response due to PHA-induced activation was smaller (22% decline) compared to lymphocytes isolated from PD animals (Figure 2A vs. Figure 2B). This effect was even more pronounced in lymphocyte Ca^2+^ release responses in the D6M population, where we observed the severest abrogation (70% decline) of the TG-induced mobilization of ER Ca^2+^ as a result of protracted elevated blood glucose (Figure 2A vs. Figure 2C). Intriguingly, at this stage of prolonged high glucose we observed only minimal increases in Ca^2+^ signals in PHA-treated lymphocytes, suggesting that the long-term high glucose condition substantially alters the capacity of the lymphocytes to adequately load ER Ca^2+^ stores.

Figure 2D,E show comparisons of the progressive loss in the TG releasable Ca^2+^ pools in unstimulated (Figure 2D) and PHA-stimulated (Figure 2E) UCD-T2DM rat lymphocyte populations at the PD, D3M and D6M time intervals. As the figures reveal, the same pattern of declination in the Ca^2+^ release response was observed in both stimulated and unstimulated lymphocyte populations, with the greatest perturbation in Ca^2+^ stores occurring at the 6-month stage of hyperglycemic diabetes (Control: PD response ΔF 0.152 ± 0.009 vs. D6M response ΔF 0.049 ± 0.005; PHA-treated: PD response ΔF 0.208 ± 0.006 vs. D6M response ΔF 0.060 ± 0.003, *p* < 0.05, *n* = 6–8, Figure 2D,E). Thus, although PHA treatment did appear to increase the levels of ER Ca^2+^ compared to unstimulated lymphocytes (Figure 2A–C), the timing and approximate scale of ER Ca^2+^ store deterioration was the same in both populations (Figure 2D vs. Figure 2E).

As mentioned above, a possible explanation for the substantial degradation in ER Ca^2+^ store content in UCD-T2DM lymphocytes is impaired ER Ca^2+^ loading due to SERCA dysfunction. Thus, we were motivated to investigate expression levels of the two major lymphocyte SERCA pump isoforms, SERCA 2b and SERCA 3 [26,27]. Figure 2F shows a representative Western blot image of SERCA 2b and SERCA 3 protein levels harvested from unstimulated or resting UCD-T2DM rat spleen lymphocytes along with the corresponding bar plots indicating gel band intensities. As the figure reveals, we observed a significant decline in expression of the SERCA 2b pump isoform at the D3M and D6M stage of diabetic hyperglycemia. And, consistent with our TG-induced Ca^2+^ release experiments (Figure 2D), we observed the greatest decline in SERCA 2b expression at the D6M stage which is likely to explain the extensive loss in ER Ca^2+^ consequent to chronic hyperglycemia. Intriguingly, we did not observe the same pattern for SERCA 3 expression. Indeed, Figure 2F shows that initially at the D3M stage of elevated blood glucose we actually observed an increase in expression levels of the SERCA 3 pump isoform. However, this augmented SERCA 3 expression effect is ultimately lost, since by the D6M stage UCD-T2DM lymphocyte SERCA 3 expression levels have significantly declined (Figure 2F). These results suggest, as we have previously reported, that the SERCA 2b and SERCA 3 Ca^2+^ pumps are linked to distinct regulatory functions in lymphocyte Ca^2+^ signaling networks [20,21]. Indeed, the initial increase in SERCA 3 expression may reflect upregulation of this Ca^2+^ transporter recruited to manage ER Ca^2+^ stores in a hyperglycemic environment subjected to ER stress; however, with continued unmanageable elevated glucose this stress response is lost and SERCA 3 protein levels eventually wane. Clearly, however, at the D6M stage of chronic elevated blood glucose we observed the maximal degree of ER Ca^2+^ store disruption, which corresponds to our observations that both SERCA 2b and SERCA 3 pump expression levels by this time interval have substantially declined.

We noted an interesting difference when we examined SERCA expression levels in lymphocytes stimulated with the T-cell mitogen PHA (Figure 2G). In contrast to unstimulated lymphocytes, when we activated T cells with PHA exposure, we observed a significant increase in SERCA 2b protein levels at the D3M stage of hyperglycemia, suggesting that T cell activation was specifically driving increased SERCA 2b expression, given that no difference was observed in the SERCA 3 expression pattern in the unstimulated vs. PHA-stimulated lymphocyte population (Figure 2F vs. Figure 2G). However, although we observed an increase in SERCA 2b expression at the D3M stage, as previously noted (Figure 2E), there was a loss in TG-releasable ER Ca^2+^ from PHA-treated lymphocytes at this stage of chronic hyperglycemia. Thus, even though lymphocytes appear to be increasing SERCA 2b expression levels it may be that there are functional impairments in SERCA activity in elevated glucose conditions, as has been previously reported in other cell types [14,15]. These results further underscore the plausibility of SERCA pump dysfunction either due to altered expression levels or functional impairments as a key factor in lymphocyte Ca^2+^ signaling defects associated with diabetic pathogenesis.

**Figure 3:**
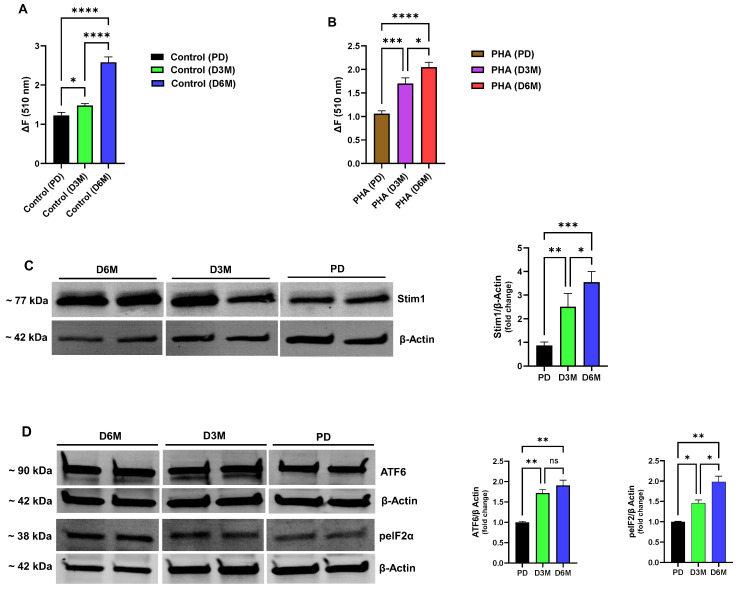
Diabetes progression in UCD-T2DM rat lymphocyte results in ER Ca^2+^ store degradation, heightened Ca^2+^ influx activation and an ER stress condition. For (**A**,**B**), lymphocytes obtained from UCD-T2DM rats were suspended in Ca^2+^-free media and Ca^2+^ release was induced by application of TG (3 μM). Subsequent to TG-induced responses, Ca^2+^ was added back to the medium (2 mM) to measure degree of Ca^2+^ influx activation. (**A**) Ca^2+^ influx responses in unstimulated lymphocytes from UCD-T2DM rats at pre-diabetic (PD, black bar), three-month diabetic (D3M, green bar) and six-month diabetic (D6M, blue bar) stages. (**B**) Ca^2+^ influx responses in PHA-stimulated lymphocytes from UCD-T2DM rats at pre-diabetic (PD, orange bar), three-month diabetic (D3M, purple bar) and six-month diabetic (D6M, red bar) stages. (**C**) Representative Western blot showing Stim1 expression levels in lymphocytes isolated from UCD-T2DM rats at the PD, D3M and D6M stages of hyperglycemia. Figure also shows bar plots of gel densitometry values determined from gel bands and presented as values normalized to β-actin expression levels. Each bar represents average band intensity value ± SEM as determined from *n* = 3–6 animals. (**D**) Representative Western blot showing ATF6 and p-eIF2α expression levels in lymphocytes isolated from UCD-T2DM rats at PD, D3M and D6M stages of hyperglycemia. Figure also shows bar plots of gel densitometry values determined from gel bands and presented as values normalized to β-actin expression levels. Each bar represents average band intensity value ± SEM as determined from *n* = 3–6 animals. Asterisks denote statistical significance with * *p* < 0.05, ** *p* < 0.005, *** *p* < 0.001 and **** *p* < 0.0001, and ns is not significant *p* > 0.05.

In addition to perturbations in ER Ca^2+^ stores, we observed a significantly altered TG-induced Ca^2+^ influx response in the UCD-T2DM rat lymphocyte population exposed to chronically elevated glucose levels (Figure 3A,B). Similar to the Ca^2+^ release responses, we observed the same general pattern of altered Ca^2+^ influx activity in both unstimulated (Figure 3A) and PHA-stimulated (Figure 3B) lymphocytes, emphasizing that the disrupted Ca^2+^ signaling landscape in the hyperglycemic condition manifests regardless of lymphocyte stimulation. These experiments revealed that with chronic hyperglycemia and progressive depletion of ER Ca^2+^ stores we observed a correspondingly increased Ca^2+^ influx response, with the greatest amplitude response occurring in the D6M lymphocyte population with the severest diminishment in Ca^2+^ store levels (Control ΔF ratio: D6M/PD = 110%; PHA-stimulated ΔF ratio: D6M/PD = 93%, *n* = 6–8, *p* < 0.05 Figure 3A,B). Thus, these results suggest that a dysfunctional lymphocyte Ca^2+^ signaling state may emerge in diabetes due to chronically depleted ER Ca^2+^ stores with an associated hyperactivated coupling signal driving inappropriate Ca^2+^ influx signals. Distortions in these key antigen-activated Ca^2+^ signaling pathways are therefore likely to result in impairments of lymphocyte function with consequent immune system deficiencies observed in the diabetic condition. Given our observation that UCD-T2DM rat lymphocytes exhibit pronounced activation of Ca^2+^ influx pathways at the 3- and 6-month hyperglycemic stage, we hypothesized that the Stim1 protein, as the major transducer of depleted ER Ca^2+^ stores, would be elevated. Indeed, we did observe a significant increase in expression of Stim1 in D3M and D6M rat lymphocytes as shown in Figure 3C (D3M: 2.5-fold increase; D6M: 3.5-fold increase). Thus, we suggest that an ER stress condition gradually develops in lymphocytes as chronic hyperglycemia progresses in part due to decreased SERCA expression/function with consequent rundown and depletion of critical Ca^2+^ levels in ER stores. Cells will likely attempt to counter or control ER Ca^2+^ loss by increasing expression of Stim1 to promote Orai1 Ca^2+^ channel activation and Ca^2+^ influx to replenish depleted stores.

In view of our experiments revealing severely depleted ER Ca^2+^ stores and upregulation of Stim1 expression as UCD-T2DM lymphocytes progress to late-stage hyperglycemia, we explored the possibility that these signaling impairments may be associated with the development of an ER stress condition. We chose two commonly investigated markers of ER stress, testing for changes in expression levels of activating transcription factor 6 (ATF6) and the phosphorylated form of eukaryotic initiation factor 2α (p-eIF2α) as a key mediator of the ER stress condition via its actions as a global protein translation inhibitor [28,29]. As shown in Figure 3D we did observe significant increases in the expression levels of ATF6 and p-eIF2α in lymphocytes experiencing chronic elevated blood glucose in the D3M and D6M phases (ATF6: D3M 1.7-fold increase, D6M 1.9-fold increase; p-eIF2α: D3M 1.4-fold increase, D6M 1.9-fold increase, Figure 3D). We did note that expression of ATF6 appeared to saturate earlier in the D3M hyperglycemic stage as compared to p-eIF2α which rose to maximal levels of expression corresponding to the D6M stage in which we observed the greatest degradation in ER Ca^2+^ levels. This temporal sequence of deployment of ER stress mediators may relate to their respective roles in ER stress transduction; ATF6 and PKR-like ER Kinase (PERK) are proximal ER resident sensors of ER stress such that their recruitment and activation occurs early in the pathway, whereas mediators like PERK-dependent p-eIF2α are downstream effectors which are likely to reach peak levels of activity later in the sequence [28,29,30]. Thus, our findings suggest that immune system dysfunction in the chronic diabetic state may occur consequent to the pronounced alterations in ER Ca^2+^ store management with aberrant Ca^2+^ influx engagement and an ensuing corrupted ER organellar state with recruitment of ER stress pathways.

### 3.2. T Lymphocytes Subjected to Long-Term High Glucose Undergo Ca^2+^ Signaling Perturbations Characterized by Reduced SERCA 2b Expression, ER Stress Induction and Loss of Cell Viability

We next shifted our studies to the use of the well-characterized model human Jurkat T cell line to take advantage of the benefits of using a clonally homogenous T cell population, thereby enabling avoidance of potentially conflicting effects likely present in a heterogeneous mix of primary spleen lymphocytes. The use of the Jurkat clonal T cell population also permits a more unambiguous assessment of the earliest changes developing in corrupted Ca^2+^ signaling pathways exposed to high glucose perturbation, given that complex cell population heterogeneity in spleen cells may mask the initial small disruptions that precede the larger overt damage observable in late-stage hyperglycemia. We established, as noted below, clear parallels between Ca^2+^ signaling alterations observed in the UCD-T2DM rat lymphocyte population and our Jurkat T lymphocyte in vitro high-glucose configuration, which provides assurance for productive use of the Jurkat T lymphocyte in these studies.

**Figure 4:**
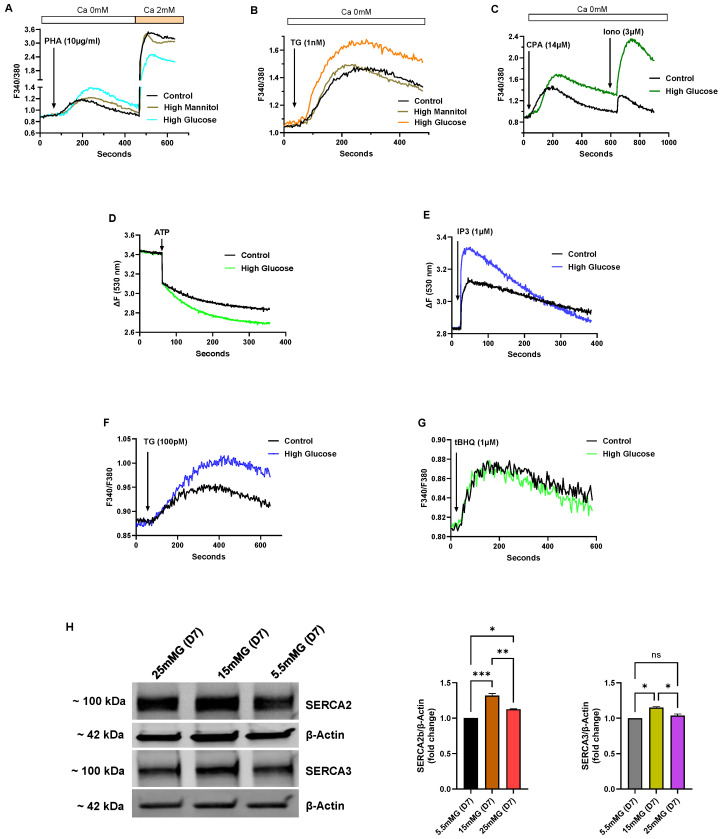
Early changes to Jurkat T cell Ca^2+^ signal regulation in response to high glucose exposure reveal enhanced ER Ca^2+^ store capacity and a preferential increase in SERCA 2b expression and activity. For (**A**–**C**), Jurkat lymphocytes were loaded with Fura-2 and suspended in Ca^2+^-free media (balanced salt solution plus 2 mM EGTA). (**A**) Jurkat cells were grown in the presence of standard medium glucose (Control, 5.5 mM, black trace), high mannitol (25 mM, brown trace) or high glucose (25 mM, blue trace) for 7 days and Ca^2+^ release was induced by application of PHA (10 μg/mL). Ca^2+^ influx responses were then initiated by adding back Ca^2+^ to the medium (2 mM). (**B**) Same experiment as described in (**A**) but Ca^2+^ release was induced by addition of TG (1 nM). (**C**) Same experiment as described in (**A**) but Ca^2+^ release was induced by application of CPA (14 μM). Ionomycin (Iono, 3 μM) was subsequently added to induce further Ca^2+^ release prior to addition of external Ca^2+^ (2 mM) to stimulate Ca^2+^ influx. (**D**) Fluo-3 fluorescence Ca^2+^ uptake assay. Ca^2+^ uptake in ER stores was initiated by addition of ATP (see Materials and Methods section) in saponin permeabilized Jurkat lymphocytes suspended in an intracellular-like medium (ICM, see Materials and Methods) and incubated in the presence of control normal glucose levels (5.5 mM, black trace) or high glucose (25 mM, green trace) for 7 days. Rate of Ca^2+^ uptake was estimated based on the linear initial rate of Fluo-3 fluorescence decay. (**E**) Experiment using saponin permeabilized Jurkat cells, depicting Ca^2+^ release responses as detected by Fuo-3 fluorescence changes. Ca^2+^ release was induced in permeabilized Jurkat cells treated with IP3 (1 μM) following seven-day incubation in presence of control normal glucose levels (5.5 mM, black trace) or high glucose levels (25 mM, blue trace). (**F**) Ca^2+^ release induced in Fura-2 loaded Jurkat lymphocytes suspended in Ca^2+^-free media by addition of TG (100 pM) in cells incubated for 7 days in Control glucose levels (5.5 mM, black trace) or high glucose levels (25 mM, blue trace). (**G**) Same experiment as in (**F**), but Ca^2+^ release was induced by addition of tBHQ (1 μM). (**H**) Representative Western blot showing expression levels of SERCA 2 and SERCA 3 from Jurkat lymphocytes incubated for seven days in control (5.5 mM), intermediate high (15 mM) and high (25 mM) glucose levels. Also shown are corresponding gel densitometry plots for SERCA 2b and SERCA 3 expression levels normalized to β-actin expression. Fluorescence traces of Ca^2+^ experiments shown are representative of three to six separate experiments with significant differences assessed via use of Student’s t test. One-way ANOVA followed by Tukey’s multiple comparison tests were used to analyze Western blot data for SERCA expression levels with *n* = 3. Asterisks denote statistical significance with * *p* < 0.05, ** *p* < 0.005, *** *p* < 0.001 and ns is not significant *p* > 0.05.

Figure 4 shows a series of experiments performed on Jurkat lymphocytes maintained in standard growth medium but with the glucose concentration increased from 5.5 mM (normal) to intermediate high (15 mM) and high (25 mM) glucose for an incubation period of 7 days, and thus denoted in our experiments as short-term high-glucose growth conditions. In select experiments, we used mannitol in place of glucose at the same concentrations to ensure that the observed effects were specific for glucose and not attributable to nonspecific osmotic effects (Figure 4A,B). Figure 4A–C depict Ca^2+^ responses elicited by various Ca^2+^ mobilizing agonists in Jurkat lymphocytes exposed to short-term (<10 days) high glucose (25 mM) in the growth medium. Figure 4A shows increased Ca^2+^ release responses induced by the TCR agonist PHA in high-glucose medium compared to PHA-induced responses in control cells (High glucose ΔF 0.52 ± 0.012 vs, Control glucose ΔF 0.31 ± 0.008, *p* < 0.05, *n* = 5). The figure also reveals diminished PHA-induced Ca^2+^ influx responses observed in high-glucose treated cells (High glucose ΔF 1.46 ± 0.015 vs. Control glucose ΔF 2.58 ± 0.02, *p* < 0.05, *n* = 5, Figure 4A), suggesting weaker ER coupling to Ca^2+^ influx pathways perhaps due to higher ER Ca^2+^ content with a corresponding attenuated depletion-activated signal. Figure 4B shows a similar response in cells treated with TG (1 nM) to achieve Ca^2+^ release via SERCA pump inhibition in short-term high glucose T lymphocytes (High glucose TG ΔF 0.62 ± 0.008 vs. Control glucose TG ΔF 0.43 ± 0.005, *n* = 5). We also used cyclopiazonic acid (CPA, 14 μM) as a structurally distinct SERCA blocker to further test ER Ca^2+^ store levels in high-glucose treated Jurkat lymphocytes (High glucose CPA ΔF 0.80 ± 0.009 vs. Control glucose CPA ΔF 0.59 ± 0.004, *n* = 3, Figure 4C). Low concentrations of the SERCA blockers TG and CPA have previously been reliably employed as Ca^2+^-releasing agents enabling assessments of ER Ca^2+^ store levels; and we show in these experiments (Figure 4B,C) that initial high glucose exposure appears to result in an early change whereby T lymphocytes re-configure ER stores to accommodate increased Ca^2+^ levels. This effect of augmented Ca^2+^ store content also appeared to apply more broadly to global Ca^2+^ storage sites in the T lymphocyte given we observed a significantly increased Ca^2+^ release response in cells treated with high concentrations of the Ca^2+^ ionophore ionomycin (High glucose ionomycin ΔF 1.05 ± 0.016 vs. Control glucose ionomycin ΔF 0.32 ± 0.003, *n* = 3, *p* < 0.05, Figure 4C), which discharges the totality of both SERCA and non-SERCA internal storage compartments.

An additional advantage of using Jurkat T lymphocytes for these studies is the ability to harvest large cell numbers from a clonal population of T cells to examine intracellular Ca^2+^ uptake/release experiments utilizing permeabilized cell experiments, and also to take advantage of our previously reported novel SERCA pharmacological approach that allows characterization of the specific roles of the SERCA 2b and SERCA 3 pumps in the T cell ER Ca^2+^ store system [20,21]. Indeed, Figure 4D shows an increased initial rate of Ca^2+^ uptake in Jurkat T lymphocytes exposed to short-term high glucose (25 mM) compared to control cells (High glucose rate ΔF/s 4.1 × 10^−3^ vs, Control rate ΔF/s 2.2 × 10^−3^, *n* = 3), consistent with our Ca^2+^ release experiments (Figure 4A–C) revealing elevated Ca^2+^ store levels. Thus, one potential underlying explanation for the high-glucose augmented store effect during short-term exposure is a quantifiable general increase in ATP-driven Ca^2+^ uptake into ER Ca^2+^ storage organelles. Notably, we further verified this heightened ER Ca^2+^ store state by observing an increased discharge of Ca^2+^ with the direct application of IP3 to permeabilized Jurkat lymphocytes exposed to short-term high glucose (High glucose IP3 response ΔF 0.50 ± 0.006 vs. Control glucose IP3 response ΔF 0.31 ± 0.004, *p* < 0.05, *n* = 3, Figure 4E).

We applied the low-dose TG and 2,5-di-(*tert* butyl)-1,4-benzohydroquinone (tBHQ) SERCA blocker regimen we previously reported [20] to show that high-glucose induced re-configuration of T cell Ca^2+^ stores appears to be mostly due to specific augmentation of SERCA 2b-regulated Ca^2+^ pools. Thus, we observed that low concentrations of TG (100 pM), which we have shown specifically discharges the SERCA 2b Ca^2+^ stores, mobilized a significantly larger increment of Ca^2+^ in T lymphocytes exposed to short-term high glucose compared to control cells (High glucose TG ΔF 0.13±0.005 vs. Control glucose TG ΔF 0.08 ± 0.003, *p* < 0.05, *n* = 4, Figure 4F). Conversely, application of low-dose tBHQ (1 μM), which preferentially targets SERCA 3-regulated stores, did not significantly alter Ca^2+^ release amplitudes in cells incubated in high glucose as compared to control cells (Figure 4G). Intriguingly, the early phase of elevated glucose, which presumably supports higher ATP production, appears to selectively enhance Ca^2+^ loading into SERCA 2b-regulated stores, suggesting differential roles of the SERCA pumps in managing the allocation of energy resources depending on the external T cell environment. Consistent with the results shown in Figure 4F, which suggests greater SERCA 2b activity in the early high-glucose condition, we observed a corresponding increase in expression of SERCA 2b protein levels (Figure 4H). Figure 4H shows a representative Western blot image along with bar plots of band intensities of SERCA 2b and SERCA 3 expression levels in Jurkat T lymphocytes exposed to normal glucose (5.5 mM), intermediate high glucose (15 mM) and high glucose (25 mM) concentrations in short-term (7 days) growth conditions. As shown, we observed a significant increase in SERCA 2b protein expression in the short-term interval in T lymphocytes exposed to intermediate glucose levels, yet expression levels start to fall, albeit still elevated, when cells are exposed to high glucose (Figure 4H). In contrast, consistent with the tBHQ-induced Ca^2+^ release experiments shown in Figure 4G, we observed a smaller change in SERCA 3 expression levels, increasing only modestly to short-term intermediate-high glucose levels and subsequently falling back to levels similar to control cells in conditions of high-glucose exposure (Figure 4H). Interestingly, the SERCA expression experiments hint at early changes occurring in T lymphocytes as the high-glucose stress environment develops; it appears as cells first encounter intermediately elevated glucose in the short-term that SERCA 2b levels are increased allowing the conditioning of ER Ca^2+^ stores to accommodate greater storage capacity. However, with exposure to the increased stressor of high glucose (25 mM), even in the short-term interval, T lymphocytes exhibit scaled-back expression of the SERCA 2b pump.

**Figure 5:**
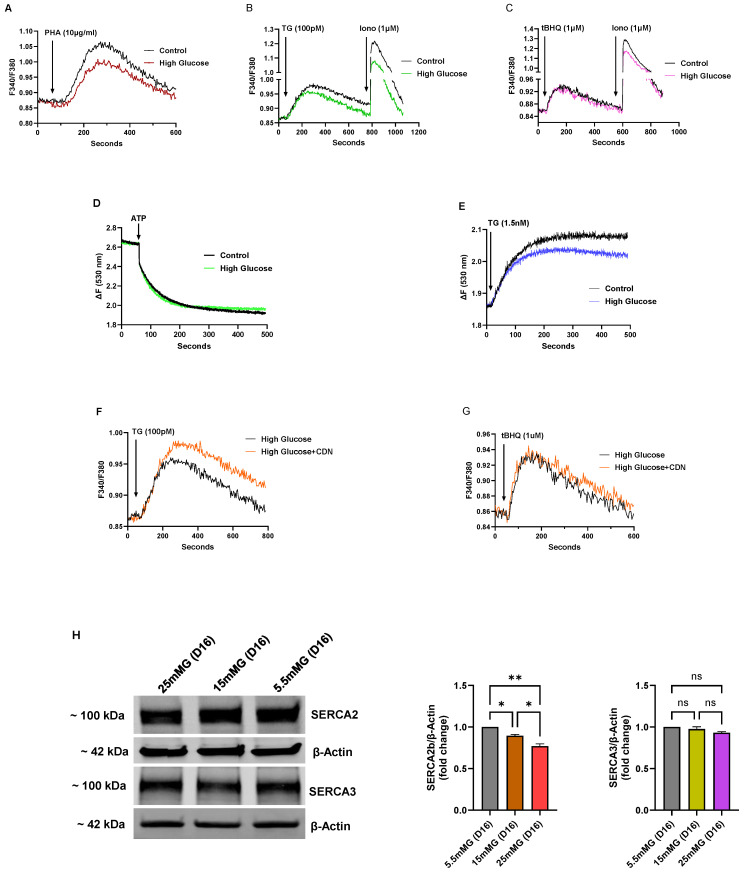
Long-term changes to Jurkat T cell Ca^2+^ signal regulation in response to high glucose exposure reveal diminished ER Ca^2+^ store capacity and selective impairment of SERCA 2b expression and activity. For (**A**–**C**), Jurkat lymphocytes were loaded with Fura-2 and suspended in Ca^2+^-free media (balanced salt solution plus 2 mM EGTA). (**A**) Jurkat cells were grown in presence of standard medium glucose (Control, 5.5 mM, black trace) or high glucose (25 mM, red trace) for 16 days and Ca^2+^ release was induced by application of PHA (10 μg/mL). (**B**) Same experiment as described in (**A**) but Ca^2+^ release was induced by the addition of TG (100 pM) and ionomycin (Iono, 1 μM) was subsequently added to induce further Ca^2+^ release. (**C**) Same experiment as described in (**B**) but Ca^2+^ release was induced by the application of tBHQ (1 μM). (**D**) Fluo-3 fluorescence Ca^2+^ uptake assay. Ca^2+^ uptake in ER stores was initiated by addition of ATP (see Materials and Methods section) in saponin permeabilized Jurkat lymphocytes suspended in an intracellular-like medium (ICM) and incubated in presence of control normal glucose levels (5.5 mM, black trace) or high glucose (25 mM, green trace) for 16 days. Rate of Ca^2+^ uptake was estimated based on the linear initial rate of Fluo-3 fluorescence decay. (**E**) Experiment using saponin permeabilized Jurkat cells, depicting Ca^2+^ release responses as detected by Fuo-3 fluorescence changes. Ca^2+^ release was induced in permeabilized Jurkat cells treated with TG (1.5 nM) following a 16-day incubation in presence of control normal glucose levels (5.5 mM, black trace) or high glucose levels (25 mM, blue trace). (**F**) Ca^2+^ release induced in Fura-2 loaded Jurkat lymphocytes suspended in Ca^2+^-free media by addition of TG (100 pM) in cells incubated for 16 days in high glucose (25 mM), followed by an additional 72 h incubation in the presence (red trace) or absence (black trace) of CDN1163 (10 μM). (**G**) Same experiment as in (**F**), but Ca^2+^ release was induced by the addition of tBHQ (1 μM). (**H**) Representative Western blot showing expression levels of SERCA 2 and SERCA 3 from Jurkat lymphocytes incubated for 16 days in control (5.5 mM), intermediate high (15 mM) and high (25 mM) glucose levels. Also shown are corresponding gel densitometry plots for SERCA 2b and SERCA 3 expression levels normalized to β-actin expression. Fluorescence traces of Ca^2+^ experiments shown are representative of three to six separate experiments with significant differences assessed via use of Student’s *t* test. One-way ANOVA followed by Tukey’s multiple comparison tests were used to analyze Western blot data for SERCA expression levels with *n* = 3. Asterisks denote statistical significance with * *p* < 0.05, ** *p* < 0.005 and ns is not significant *p* > 0.05.

As the Jurkat T lymphocytes are subjected to long-term high-glucose exposure (16 days, 25 mM) we observed similar effects on ER Ca^2+^ stores as was noted for hyperglycemic UCD-T2DM rats. We observed, for example, significantly reduced TCR-activated Ca^2+^ release (using the TCR agonist PHA) in T lymphocytes exposed to long-term (>10 days) high glucose (High glucose ΔF 0.138 ± 0.008 vs. Control glucose ΔF 0.195 ± 0.005, *p* < 0.05, *n* = 5, Figure 5A), reversing the augmented ER Ca^2+^ effect we noted in short-term high glucose conditions (Figure 4A). Similarly, we also observed an attenuated Ca^2+^ release response inducible by low-dose TG (100 pM), suggesting, as noted above, that the primary perturbatory action of high-glucose is directed to the SERCA 2b Ca^2+^ pump (High glucose TG ΔF 0.097 ± 0.005 vs. Control glucose TG ΔF 0.120 ± 0.003, *p* < 0.05, *n* = 5, Figure 5B); accordingly, as with the short-term high-glucose effect, we did not find a significant alteration of the low-dose tBHQ-stimulated Ca^2+^ release response (Figure 5C), again suggesting that the SERCA 3-regulated Ca^2+^ stores may be relatively unaffected by changes in glucose levels for these incubation periods. Interestingly, although our low-dose SERCA blocker (TG and tBHQ) experiments indicate differential sensitivities of SERCA-regulated Ca^2+^ pools, we did observe a more global diminishment of Ca^2+^ storage reservoirs as revealed by ionomycin application (1 μM, Figure 5B,C), similar to the effects we noted above in rat lymphocytes subjected to chronic hyperglycemia.

Using the permeabilized lymphocyte assay to examine Ca^2+^ uptake and release effects directly, we observed reduced ER Ca^2+^ uptake back to levels indistinguishable from control normal glucose levels (Figure 5D vs. Figure 4D), and an attenuated permeabilized cell TG (1.5 nM)-induced ER Ca^2+^ release response in Jurkat lymphocytes exposed to long-term high-glucose conditions (High glucose TG ΔF 0.18 ± 0.007 vs. Control glucose TG ΔF 0.23 ± 0.004, *n* = 3, Figure 4E). These results suggest, as we noted using the UCD-T2DM rat lymphocytes, an impairment in SERCA-mediated Ca^2+^ ER loading, likely due to high-glucose induced perturbations affecting ER energy homeostasis with an ensuing deficiency to sequester Ca^2+^ into storage compartments. Intriguingly, we observed that incubation with the SERCA activator CDN1163 (10 μM, 72 h) significantly boosted Ca^2+^ release responses induced by low-dose TG (100 pM) in Jurkat lymphocytes exposed to long-term (16 days) high glucose (TG/CDN1163 ΔF 0.126 ± 0.007 vs. TG/no CDN1163 ΔF 0.097 ± 0.005, *p* < 0.05, *n* = 4, Figure 5F), which as we have previously reported specifically targets the SERCA 2b Ca^2+^ pools [20]. In contrast, Figure 5G shows that CDN1163 treatment did not have a similar effect in restoring low-dose tBHQ responses in the long-term high-glucose condition, consistent with our short-term high-glucose experiments (Figure 4F,G), suggesting differential effects of high-glucose perturbations on SERCA 2b vs. SERCA 3 Ca^2+^ stores. This finding adds to previous studies noting beneficial effects of pharmacological SERCA activation in cardiovascular pathologies [31,32,33,34], with our work now finding evidence that a protective effect of CDN1163 operates via preferential augmentation of SERCA 2b-mediated Ca^2+^ loading in T cell Ca^2+^ stores. This pharmacological effect is also consistent with our SERCA expression studies showing that the initial T cell response to short-term high glucose is to transiently increase the SERCA 2b Ca^2+^ pump which appears to result in increased ER Ca^2+^ storage capacity (Figure 4H). Consistent with this idea we show in Figure 5H that with continued long-term high-glucose exposure the boost in SERCA 2b Ca^2+^ pump expression is reversed, revealing a progressive loss of expression of the transporter with intermediate and high glucose extended exposure. Moreover, as noted previously, expression and function of the SERCA 3 Ca^2+^ pump isoform appears less sensitive to perturbations induced by T cell growth in high-glucose conditions (Figure 5H). These findings further underscore the potential value of therapeutic targeting of SERCA 2b Ca^2+^ pumps to protect ER Ca^2+^ stores, and thereby preserve T cell function in a hyperglycemic environment.

**Figure 6:**
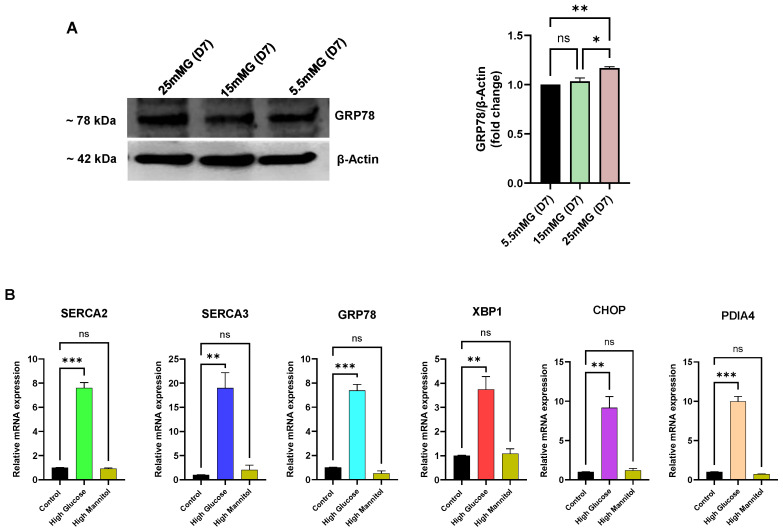
High glucose exposure induces SERCA pumps and core ER stress mediators in Jurkat lymphocytes that precede detectable changes in agonist-induced ER Ca^2+^ release. (**A**) Representative Western blot showing GRP78 expression levels in Jurkat lymphocytes exposed to normal (5.5 mM), intermediate high (15 mM) and high (25 mM) glucose levels for 7 days. Also shown is gel densitometry plot depicting changes in GRP78 levels normalized to β-actin expression levels in normal (5.5 mM, black bar), intermediate high (15 mM, green bar) and high (25 mM, pink bar) glucose levels. (**B**) RT-qPCR experiments performed on Jurkat lymphocytes incubated in high glucose (25 mM) for 3 days depicting relative mRNA levels for SERCA 2, SERCA 3, GRP78, XBP1, CHOP and PDIA4. Also shown are control experiments of cells grown in normal (5.5 mM) glucose levels and mannitol (25 mM). All mRNA levels were normalized to control GAPDH mRNA. One-way ANOVA followed by Tukey’s and Dunnett’s multiple comparison tests were used to analyze Western blot data for GRP78 expression levels and RT-qPCR data with *n* = 3, respectively. Asterisks denote statistical significance with * *p* < 0.05, ** *p* < 0.005, *** *p* < 0.001 and ns is not significant *p* > 0.05.

Given that we observed an apparent deterioration in ER Ca^2+^ store-loading capacity in Jurkat lymphocytes exposed to long-term (>10 days) high glucose, we sought to examine whether these Ca^2+^ store perturbations associated with an elevated ER stress state, as we noted in the UCD-T2DM rat lymphocytes. Indeed, as shown in Figure 6 we did observe a significant increase in key ER-resident markers of the ER stress condition. Figure 6A shows, for example, increased expression of the major ER chaperone and ER-stress regulator protein GRP78. This experiment revealed, as we noted in Figure 4H for declining SERCA expression levels, early changes (<10 days) in expression levels with increasing GRP78 production by day 7 in Jurkat lymphocytes exposed to high glucose (Figure 6A), preceding the measurable loss in ER Ca^2+^ levels detectable in our long-term high-glucose condition. Similarly, Figure 6B shows RT-qPCR experiments measuring elevation of mRNA levels of an expanded list of common ER stress markers and SERCA pumps within our short-term (<10 day) high-glucose exposure interval, reflecting early gene expression changes in Jurkat lymphocytes to high-glucose perturbations in ER conditions that appear before our detection of diminished ER Ca^2+^ store content (>10 days, Figure 5A,B). Indeed, in alignment with our protein expression studies (Figure 4H) we noted an increase (3 days) in mRNA levels for SERCA 2b (7-fold) and SERCA 3 (17-fold), likely reflecting an early adaptive response to preserve ER Ca^2+^ store levels in response to high external glucose concentrations. In addition to upregulation of the ER chaperone and protein folding mediators GRP78 (6-fold increase) and Protein Disulfide Isomerase A4 (PDIA4, 9-fold increase), we observed increased expression of the genes encoding key transcriptional regulators and effectors of the ER stress state in X-Box Binding Protein 1 (XBP1, 2.5-fold increase) and C/EBP Homologous Protein (CHOP, 8-fold increase). Thus, these experiments reveal that our in vitro Jurkat lymphocyte high-glucose model exposes early changes in gene and protein expression patterns, manifesting before observable alterations in Ca^2+^ release responses, that signal an impairment in ER functional integrity, likely due to disruptions secondary to exposure to high glucose conditions.

**Figure 7:**
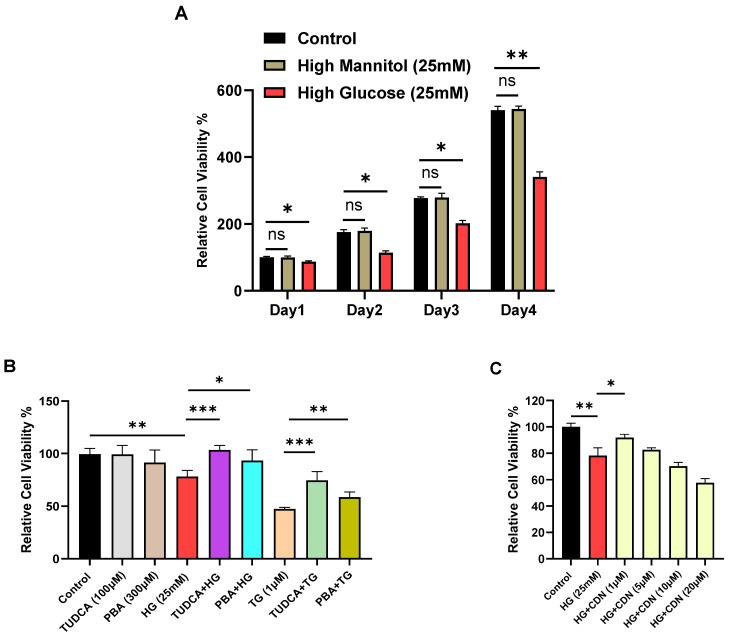
Exposure to high glucose impairs Jurkat lymphocyte cell viability that can be restored by treatment with ER chemical chaperones or SERCA activation. (**A**) Measurable loss in cell viability appears by day 4 in Jurkat lymphocytes incubated in high glucose (25 mM) as compared to untreated control cells or cells incubated in mannitol (25 mM). For analysis of viability data two-way ANOVA with repeated measures followed by Sidak’s multiple comparisons test was used. (**B**) Figure shows significant recovery to high glucose (25 mM) or TG (1 μM) of cell viability in Jurkat lymphocytes pre-treated with TUDCA (100 μM, 24 h). Figure also shows improvement of Jurkat lymphocyte viability to high glucose and TG when cells are pre-treated with PBA (300 μM, 24 h). Comparisons utilized the unpaired two-tailed Student’s *t*-test. (**C**) Dose–response effect of CDN1163 to protect against loss of cell viability in Jurkat lymphocytes exposed to high glucose (25 mM). Jurkat lymphocytes were maintained in high glucose for 14 days followed by treatment with various concentrations of CDN1163 for 72 h. Statistical analysis was by one-way ANOVA followed by Dunnett’s multiple comparison test. Asterisks denote statistical significance with * *p* < 0.05, ** *p* < 0.005, *** *p* < 0.001 and ns is not significant *p* > 0.05.

Corruption of ER function due to external high-glucose with recruitment of ER stress mediators GRP78, XBP1 and CHOP might reasonably be expected to exert an observable growth suppression effect on T lymphocytes to allow for corrective protein folding reactions and restoration of ER integrity. We thus proceeded to examine indices of cell viability in Jurkat lymphocytes subjected to high glucose. Figure 7A shows a gradual loss on viability for Jurkat lymphocytes exposed to high glucose (25 mM) with clear growth inhibition observed as early as day 4. Next, we investigated the effects of the small molecule chemical chaperones tauroursodeoxycholic acid (TUDCA) and 4-phenylbutyrate (PBA), which have been shown to provide protection against damaging ER stress associated with metabolic dysfunction, insulin resistance and type 2 diabetes [35]. Indeed, we observed that pre-incubation of Jurkat lymphocytes with TUDCA (100 μM, 24 h) and PBA (300 μM, 24 h) provided significant protection against high-glucose induced cell viability degradation occurring by day 4, largely restoring cell growth to levels similar to control lymphocytes (Figure 7B). These experiments are in alignment with the well-established property of TUDCA and PBA to mitigate ER stress [35], and our experiment also shows the capacity of these chemical chaperones to protect against the effects of TG, a well-known potent inducer of ER stress (Figure 7B). Figure 7C shows that incubation of Jurkat lymphocytes with the SERCA activator CDN1163 also provided protection against long-term (14 days) high-glucose induced loss of cell viability, revealing maximal preservation of lymphocyte cell viability at the 1–5 μM range. Importantly, this result suggests that the effects of CDN1163 to restore Ca^2+^ levels in SERCA 2b-regulated stores in lymphocytes exposed to long-term high glucose (Figure 5F) may be a valuable therapeutic strategy to protect T cell functions and viability in the presence of chronic high glucose.

## 4. Discussion

In this study we have used the UCD-T2DM type 2 diabetes rat model for an investigation of lymphocyte functional defects that may underlie immune system dysfunction. Several previous studies have found SERCA Ca^2+^ pump impairments in diabetes associated with ER stress and metabolic disruptions in liver tissue, pancreatic β cells, cardiac myocytes and endothelial cells [14,15,31]. Although immune function defects in diabetes are well-documented [1], the potential role of SERCA Ca^2+^ pump dysfunction in contributing to ER signaling disruptions in lymphocytes—an early event preceding immune system impairment—remains less well understood. Here we have found that progressive chronic hyperglycemia in lymphocytes isolated from the spleen of UCD-T2DM rats of 3–6 months reveal severely depleted ER Ca^2+^ stores. Moreover, we observed reduced SERCA 2b expression in the resting spleen lymphocyte population at 3 months of hyperglycemia (D3M), although we did note that TCR stimulation could induce increased SERCA 2b expression at this stage, yet with continued 6-month hyperglycemia (D6M), this ability to stimulate SERCA 2b upregulation is lost. In contrast, the SERCA 3 Ca^2+^ pump isoform appears to be initially increased in the resting rat lymphocyte population at the D3M stage with the cells also losing the ability to increase SERCA 3 expression by D6M in either the stimulated or resting state. Intriguingly, this observation suggests differential SERCA regulation/function in lymphocytes responding to a chronic hyperglycemic stress. In this context, it is interesting to note that SERCA 3 has previously been observed to physically interact with Stim1 to increase Ca^2+^ stores, and that this interaction was abrogated in platelets isolated from patients with type 2 diabetes [36]. Thus, this putative SERCA 3 specific effect as well as the apparent TCR-specific action on SERCA 2b-inducible expression further underscore differential roles of the SERCA pumps in T cell adaptive responses to chronic hyperglycemia.

Notably, we found that the extensive ER Ca^2+^ store depletion in lymphocytes subjected to chronic hyperglycemia correlated with enhanced Ca^2+^ influx responses. Indeed, this lymphocyte phenotype may be an important component of lymphocyte dysfunction, given an unbalanced or disproportionate ER Ca^2+^ store depletion coupled with aggressive Ca^2+^ influx could destabilize antigen-induced signaling pathways producing deficient T-cell guided immune responses. In fact, this is one possible explanation for inadequate immune responses as has been observed in the tumor microenvironment, a phenomenon whereby excessive Ca^2+^ influx drives high-level NFAT activity resulting in the T cell exhaustion phenotype and insufficient tumor immune responses [37,38]. In association with the enhanced Ca^2+^ influx observed in lymphocytes isolated from UCD-T2DM rats, we also detected increased expression of Stim1—the ER Ca^2+^ sensor activated by store depletion—suggesting a compensatory mechanism driving Ca^2+^ entry in response to reduced ER Ca^2+^ levels This finding appears to parallel the D3M increase in SERCA 3 expression noted above, and may represent an induced mechanism pairing SERCA 3 and Stim1 upregulation to replenish Ca^2+^ stores. This idea suggests a putative dual mechanism for Stim1 function in activating SERCA to increase ER Ca^2+^ uptake while also increasing Ca^2+^ influx pathways via recruitment and activation of plasma membrane-residing Orai1 Ca^2+^ channels. Thus, the Stim1–Orai1 Ca^2+^ influx pathway may be an important component of the ER stress response to restore ER Ca^2+^ stores, as supported by a recent finding linking ER stress mediators to Stim1 activation [39]. Indeed, Stim1 levels in contrast to SERCA 3 remained elevated in the extended hyperglycemic interval (D6M), consistent with the hyper-activated Ca^2+^ influx responses T lymphocytes appear to deploy to manage ER Ca^2+^ store perturbations under high-glucose stress. These findings suggest that the ER is under stress, as evidenced by the upregulation of two key ER stress markers, ATF6 and phosphorylated eIF2α All of the foregoing assessments of splenic lymphocytes obtained from diabetic UCD-T2DM rats display characteristics of immune cells with impaired signaling functions and deteriorating ER functional integrity, which are likely to manifest in deficiencies in immune function. In this context, it is interesting to note a recent study reporting a connection between reduced SERCA function and impaired recombination of antigen receptor VDJ genes in developing lymphocytes [40], representing an additional possible explanation for deficient immune competence secondary to diabetes.

We transitioned in our study to the use of the commonly employed model of T cell function, the human Jurkat T lymphocyte model [20,21,25,41,42]. Jurkat lymphocytes as a clonally homogeneous T lymphocyte allows a more granular assessment of early small changes occurring in the cell exposed to high glucose, that may be masked by a complex mix of primary heterogeneous lymphocytes. Indeed, we noted a progression of T cell signaling changes similar to effects we observed in the primary lymphocyte population of UCD-T2M rats; yet, we were also able to add additional detail to the early effects (<10 days) of high glucose exposure that suggest adaptive changes to this ER stress environment. We were able to capture, for example, that T cells increased expression of the SERCA 2b pump as glucose levels were increased in the short-term to intermediate-high levels (15 mM), but with exposure to higher glucose levels (25 mM) SERCA 2b pump expression began to fall, albeit remaining modestly elevated. In contrast, SERCA 3 pump expression also increased in the Jurkat lymphocyte to early elevated glucose, but much less prominently compared to SERCA 2b levels. Correlated with increased SERCA pump expression to short-term high-glucose exposure, we observed that T lymphocyte ER Ca^2+^ store remodeling appeared to occur given Ca^2+^ release experiments induced by TCR activation and SERCA blockade revealed increased Ca^2+^ store capacity. We were then able to use our Jurkat T lymphocyte low-dose TG/tBHQ SERCA blocker experiment to reveal that the SERCA 2b-regulated Ca^2+^ stores appeared to be primarily responsible for these early adaptive alterations to ER Ca^2+^ stores, a finding consistent with the changes we observed to the expression profile of the SERCA 2b pump. Previous investigations have also reported an intriguing bias to the actions of the SERCA 2b Ca^2+^ pump isoform in managing ER stress responses to a high-glucose metabolically altered environment [29,43].

However, with continued long-term (>10 days) high-glucose exposure the Jurkat T lymphocytes exhibited an erosion in ER Ca^2+^ store function that appeared to mimic our observations in splenic lymphocytes obtained from UCD-T2DM rats. We noted, for example, a significant drop in SERCA 2b expression levels in the longer-term high glucose environment, as the cells appeared to lose the ability to upregulate SERCA expression that was observed in the early response to intermediate-high levels of glucose. Notably, SERCA 3 pump expression remained largely unchanged following prolonged high-glucose exposure, further supporting the notion that SERCA 2b is the primary isoform involved in ER Ca^2+^ store adaptation under these conditions. Intriguingly, as noted above, we observed a substantial drop in SERCA 3 expression in the lymphocyte population isolated from the spleens of UCD-T2DM rats with chronic hyperglycemia (D6M), which may reflect a change induced by a much more prolonged high-glucose exposure or also may simply reflect comparisons between a heterogeneous population of lymphocyte phenotypes and the clonally homogeneous Jurkat lymphocyte. The Jurkat lymphocyte may represent, for example, a particular T cell phenotype with constitutive SERCA 3 expression/function and less responsivity to ER stress conditions when compared to the mix of lymphocyte phenotypes isolated from the spleen population. Indeed, studies have shown that Jurkat lymphocytes represent a clonal T cell population with constitutive phosphoinositide metabolism unlinked to TCR activation, which necessarily limits direct comparisons of Jurkat T cells with primary lymphocytes [41].

In keeping with the depressed expression levels of the SERCA pumps, and similar to our findings in the UCD-T2DM rat model, we observed diminished ER Ca^2+^ levels in Jurkat lymphocytes exposed to longer-term high glucose, as revealed by Ca^2+^ release in intact and permeabilized cells treated with TCR agonists or SERCA blockers. We employed the low-dose TG/tBHQ protocol to further demonstrate that SERCA 2b-sensitive Ca^2+^ stores are the primary targets of high-glucose–induced modulation. Specifically, very low concentrations of TG (100 pM), which preferentially inhibit SERCA 2b, elicited significantly reduced Ca^2+^ release responses, whereas low concentrations of tBHQ, that preferentially target SERCA 3, produced responses comparable to those in control cells. Moreover, we used this assay in concert with the SERCA activator CDN1163 which we have previously reported specifically increases SERCA 2b Ca^2+^ store levels [20], to reveal that treatment with a SERCA 2b activator can restore ER Ca^2+^ levels and presumably preserve ER integrity by acting on this pump isoform. Previous work has reported similar effects on Ca^2+^ signaling in Jurkat lymphocytes exposed to high glucose [44,45]; our study now adds further details on these investigations revealing novel interrelationships and relative levels of activity of the two major SERCA pump isoforms in Jurkat and primary rat spleen lymphocytes.

Consistent with our approach in the experiments performed in hyperglycemic UCD-T2DM rats, we were prompted to examine whether high-glucose conditions and altered Ca^2+^ signaling properties manifest in observable changes to Jurkat lymphocyte ER integrity and cell viability. Accordingly, we observed an incremental increase of expression levels of the major ER stress regulator GRP78 in intermediate and high-glucose environments. We also noted significant increases in gene expression levels of a conventional suite of ER stress mediators GRP78, XBP1, CHOP and PDIA4 in Jurkat lymphocytes treated with high glucose (25 mM). It is interesting to note that these ER stress mediators appear to be increasing (<10 days) prior to our detection of abrogated ER Ca^2+^ release. It may be, for example, that the early upregulation in SERCA 2b expression we observed for high glucose represents, along with induction of the ER stress mediators, a concerted program activated to protect ER Ca^2+^ stores, deployed quickly in response to the stress condition of high glucose. Indeed, we observed further changes to T lymphocyte function that signify early deleterious effects of high glucose, as we found a gradual loss of cell viability with clear suppression of cell growth as early as 4 days high-glucose exposure, which is also consistent with the time-frame we noted for increased expression of the ER stress mediators. Importantly, we were able to observe significant protection of cell viability by treating Jurkat lymphocytes with chemical chaperones TUDCA and PBA, which both have been previously shown to be effective at protecting against ER stress-induced cell damage and death [35]. And, in keeping with our finding that the SERCA activator CDN1163 preserved SERCA 2b Ca^2+^ stores, we found that the compound could also exert a preservative effect on T lymphocyte viability. Work by others has also revealed salutary effects of the SERCA activator CDN1163 in reversing cellular and tissue damage associated with ER stress in the metabolic and diabetic disrupted state [31,46,47,48]. Indeed, our study now adds to these earlier reports and highlights a potential significant therapeutic value of targeting SERCA 2b pumps for preservation of T cell and immune system functional integrity to counter diabetic pathology.

We note that this work represents a preliminary effort to investigate effects of chronic hyperglycemia on lymphocyte and immune function. Future work will continue to refine the analysis of SERCA defects and ER signaling aberrations as they relate to broader immune function in the UCD-T2DM rat hyperglycemic population. We also recognize a subset of limitations encountered in the present study. Our future work will aim to strengthen the investigation by including the nondiabetic Sprague Dawley rat as the healthy control in these experiments. It is important to acknowledge, however, that there are challenges and complications in identifying suitable animal controls for the UCD-T2DM population given tangible disparities in genetic and phenotypic characteristics that preclude optimum usage as controls for these studies. In addition, our future work will expand our investigation by inclusion of female UCD-T2DM rats to assess lymphocyte functional parameters that may be attributable to sex differences. Our future efforts will also benefit from experiments using clearly defined primary lymphocyte populations such as interrogation of hyperglycemic effects on specific T cell subsets or the B lymphocyte population, which would allow the work to move beyond the limitations presented by reliance on the Jurkat leukemic cell line. This line of investigation can also be extended in future studies to examine in more detail SERCA functional disruptions, ER stress signaling and the relationship to recruitment of the Stim1/Orai1 Ca^2+^ influx pathways and the interplay of these events in the communication between the ER and mitochondria given these key inter-organelle pathways may also be disrupted in the diabetic state.

## 5. Conclusions

We report in this study that lymphocytes isolated from UCD-T2DM rats exposed to chronic hyperglycemia reveal a progressive loss of ER Ca^2+^ store capacity which also appears correlated with pronounced alterations in SERCA Ca^2+^ pump expression levels. These findings suggest that diabetic hyperglycemia results over a period of sustained elevated glucose in degradation of SERCA expression and function with depleted ER Ca^2+^ stores, disproportionate activation of Ca^2+^ influx and a generalized dysfunctional ER stress condition as determined by the deployment of key mediators of the ER stress pathway. Thus, we suggest that these diabetic-induced changes in lymphocyte signaling functions likely underlie the gradual deterioration of immune competence known to occur in T2DM patients. We pursued further clarification of the early effects of chronic high glucose on T cell signaling functions by utilizing the well-characterized Jurkat T lymphocyte model cell line. These additional experiments using the clonal homogeneous Jurkat lymphocyte revealed intriguing SERCA isoform-specific levels of adaptive regulation of the high-glucose induced ER stress condition. Indeed, we observed that the SERCA 2b-regulated Ca^2+^ pool demonstrates greater sensitivity to high-glucose ER stress, relative to SERCA 3-regulated pools, alternately increasing followed by decreasing activity to the early/late phase exposure to high glucose. These experiments also revealed that the SERCA activator CDN1163, which we have previously shown exerts a preferential action on Jurkat T cell SERCA 2b, reversed chronic high glucose ER Ca^2+^ loss and conferred protection against cell viability damage, suggesting a potential therapeutic strategy targeting SERCA 2b pumps to preserve immune function in T2DM.

## Data Availability

The original contributions presented in this study are included in the article/Supplementary Material. Further inquiries can be directed to the corresponding author.

## Supplementary Materials

The following supporting information can be downloaded at: https://www.mdpi.com/article/10.3390/biom15070987/s1, Western blot original images.
