# Supplementary figures and images for: High Glucose in Diabetic Hyperglycemia Perturbs Lymphocyte SERCA-Regulated Ca2+ Stores with Accompanying ER Stress and Signaling Dysfunction
Source: Biomolecules. 2025 Jul 11;15(7):987. doi: 10.3390/biom15070987 (PMC12292806; doi:10.3390/biom15070987)

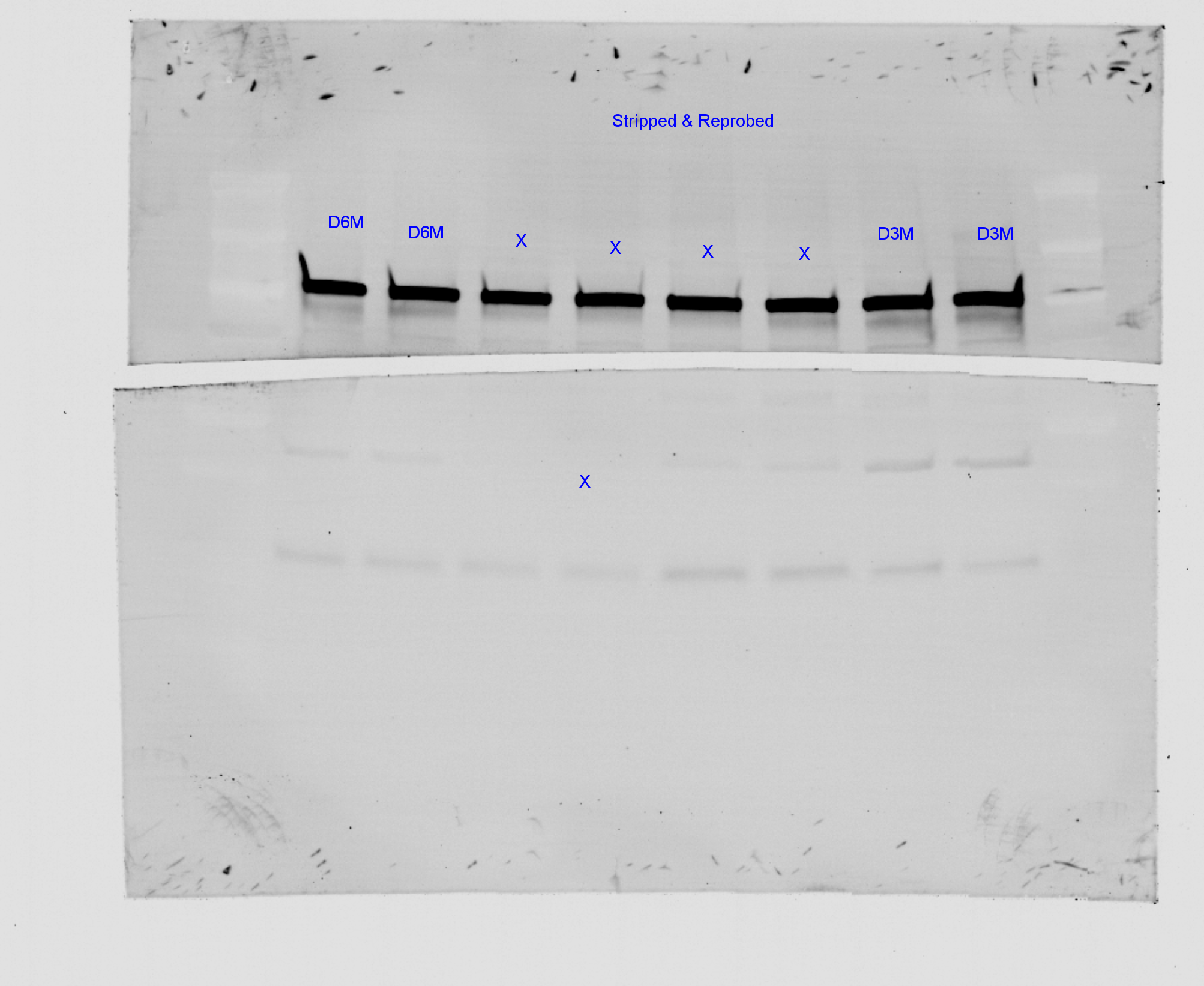

Supplement: Supplementary file 1 [file biomolecules-15-00987-s001.zip › Image_ ATF6 DM Rats.tif]

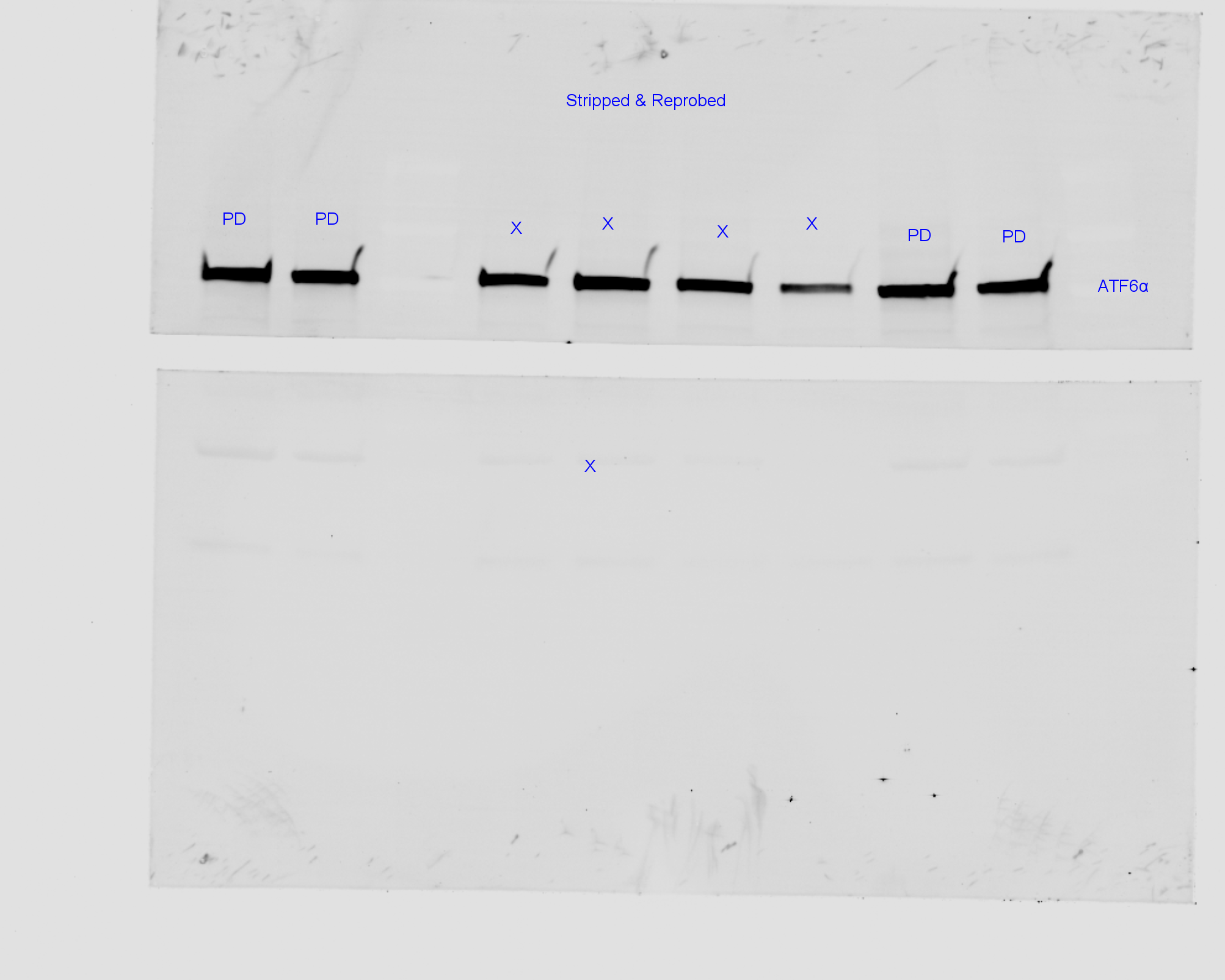

Supplement: Supplementary file 1 [file biomolecules-15-00987-s001.zip › Image_ ATF6 PD Rats.tif]

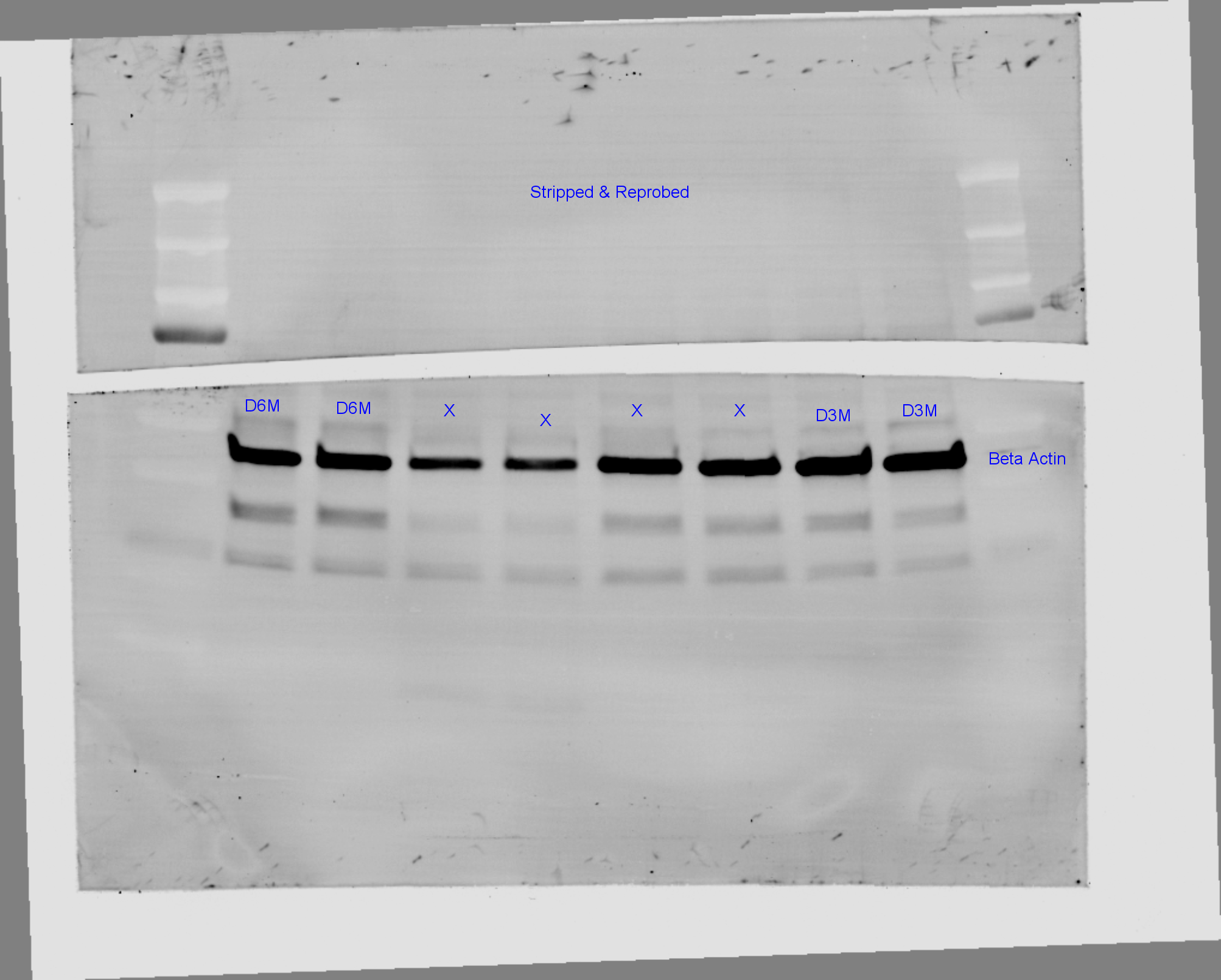

Supplement: Supplementary file 1 [file biomolecules-15-00987-s001.zip › Image_ Beta Actin for DM Rats.tif]

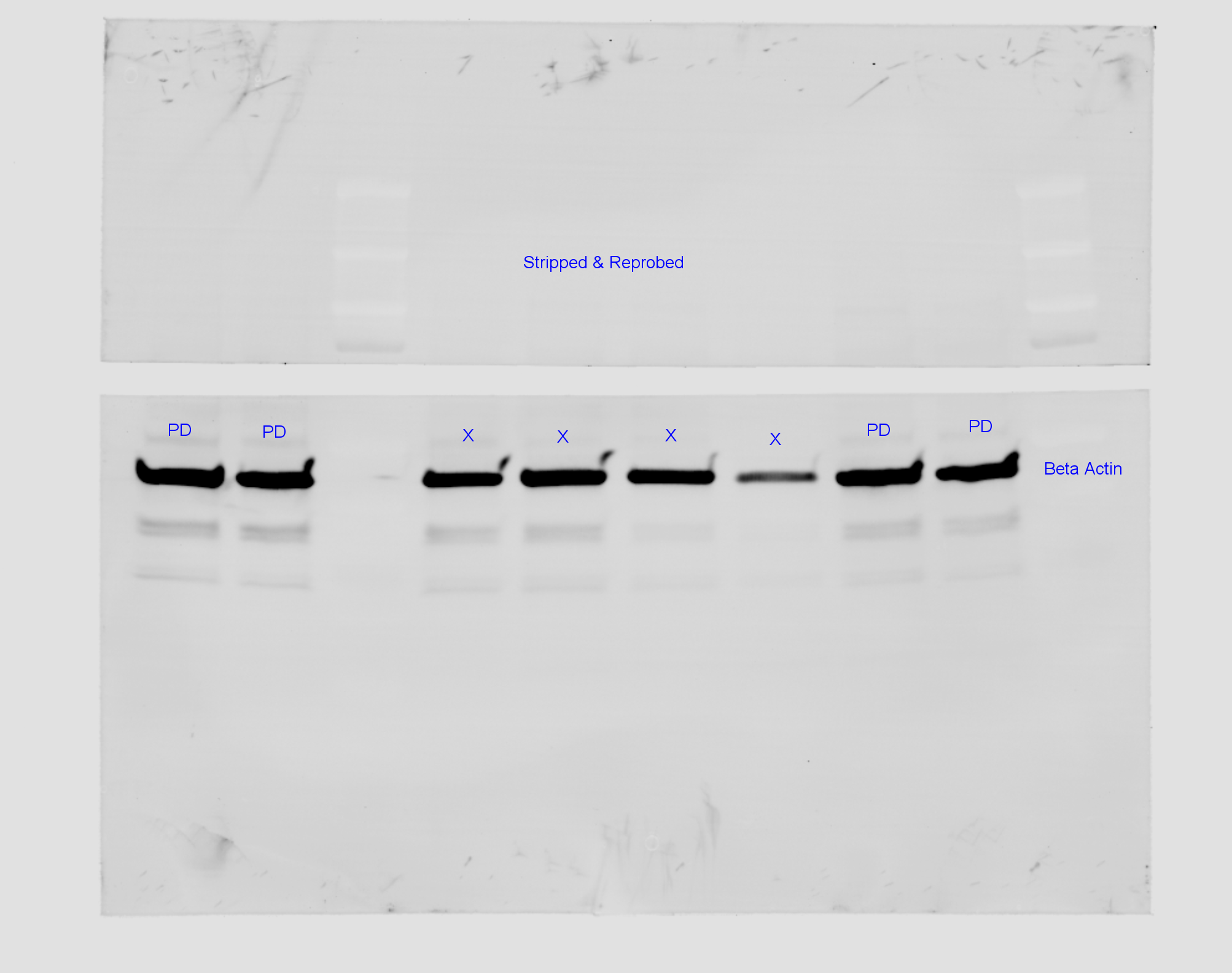

Supplement: Supplementary file 1 [file biomolecules-15-00987-s001.zip › Image_ Beta Actin for PD Rats.tif]

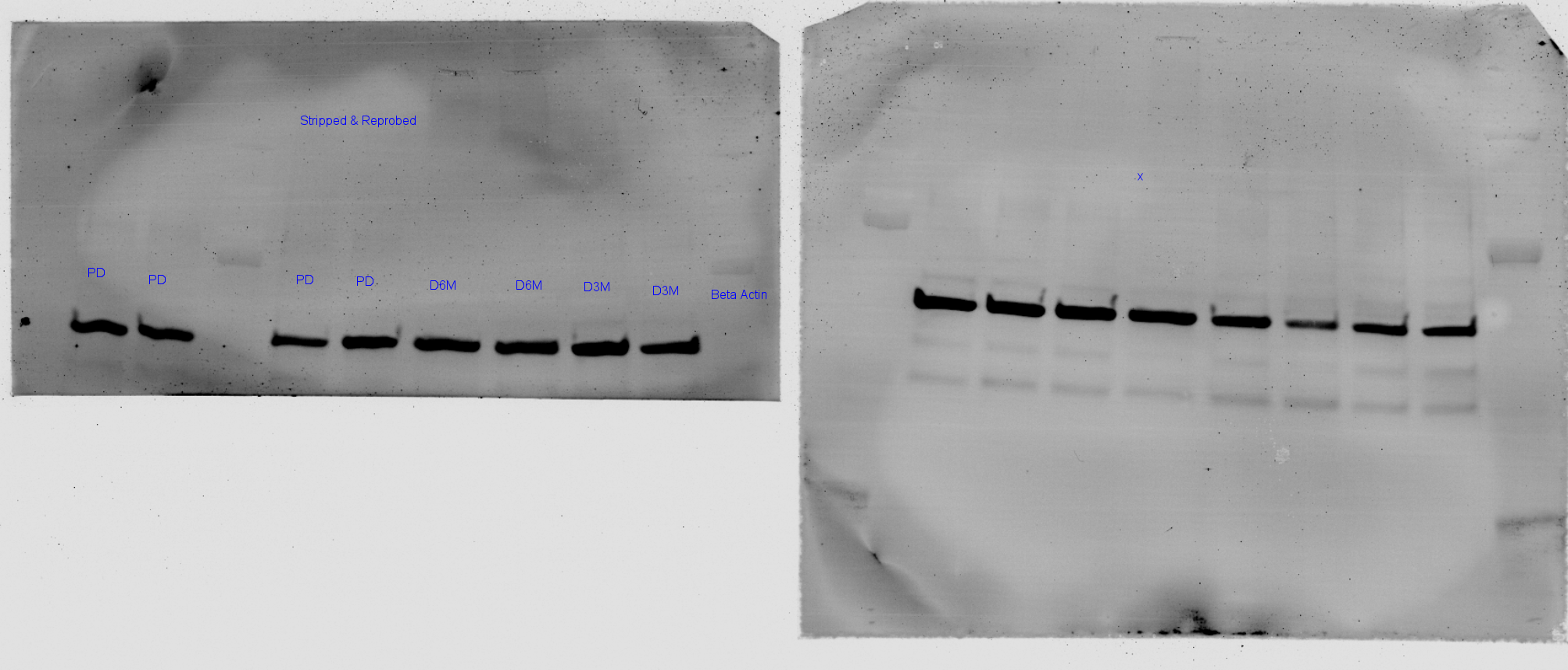

Supplement: Supplementary file 1 [file biomolecules-15-00987-s001.zip › Image_ Beta Actin for SERCA2 PHA Activated Lymphocytes PD to DM Rats.tif]

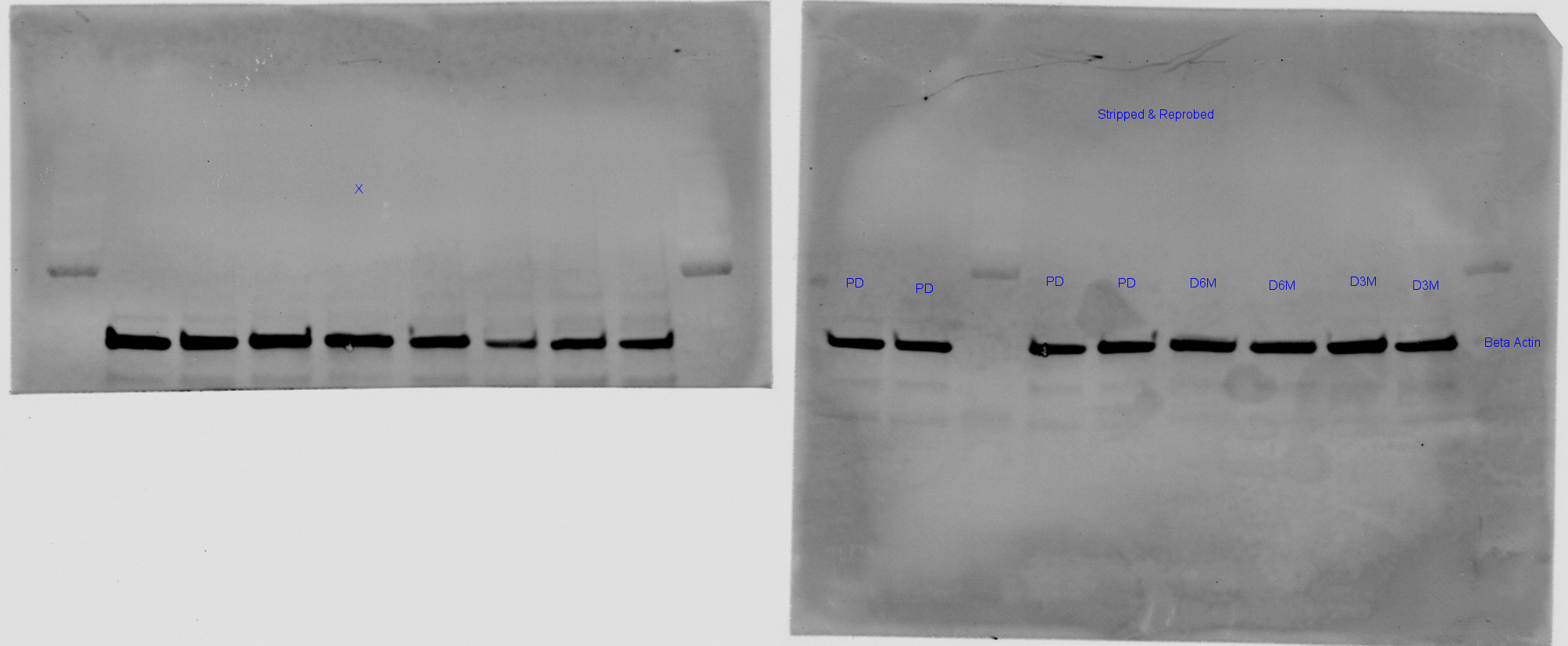

Supplement: Supplementary file 1 [file biomolecules-15-00987-s001.zip › Image_ Beta Actin for SERCA3 PHA Activated Lymphocytes PD to DM Rats.tif]

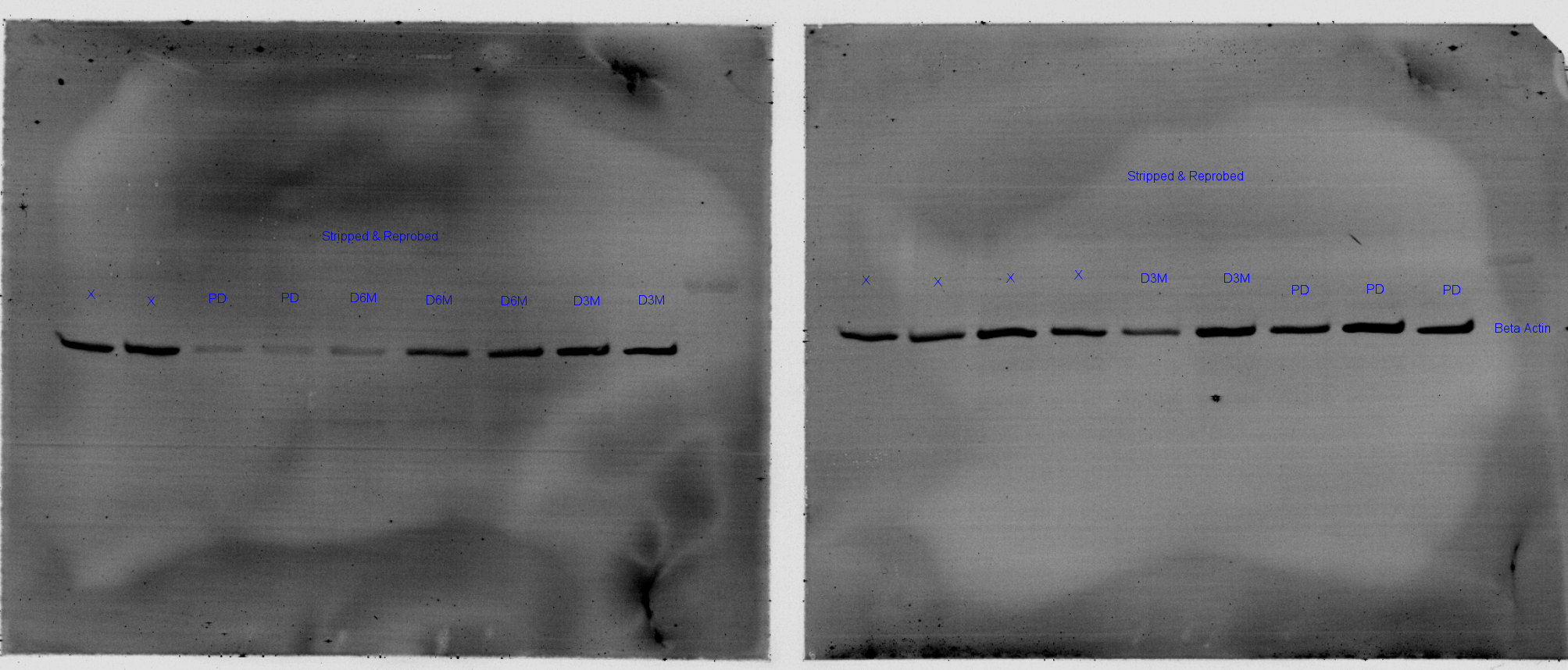

Supplement: Supplementary file 1 [file biomolecules-15-00987-s001.zip › Image_ Beta Actin for Stim1 PD to DM Rats.tif]

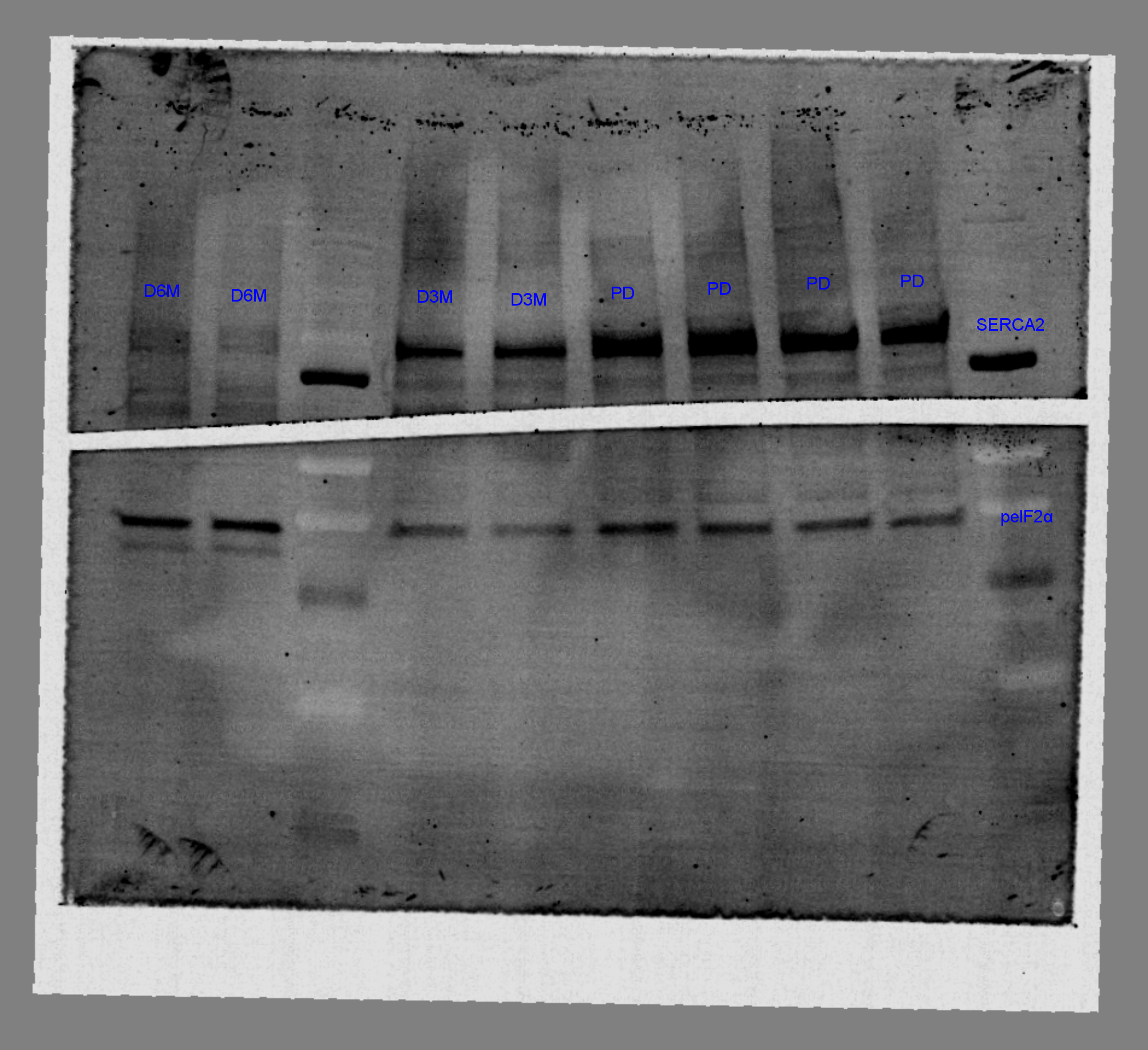

Supplement: Supplementary file 1 [file biomolecules-15-00987-s001.zip › Image_ peIF2 and SERCA2 PD to DM Rats.tif]

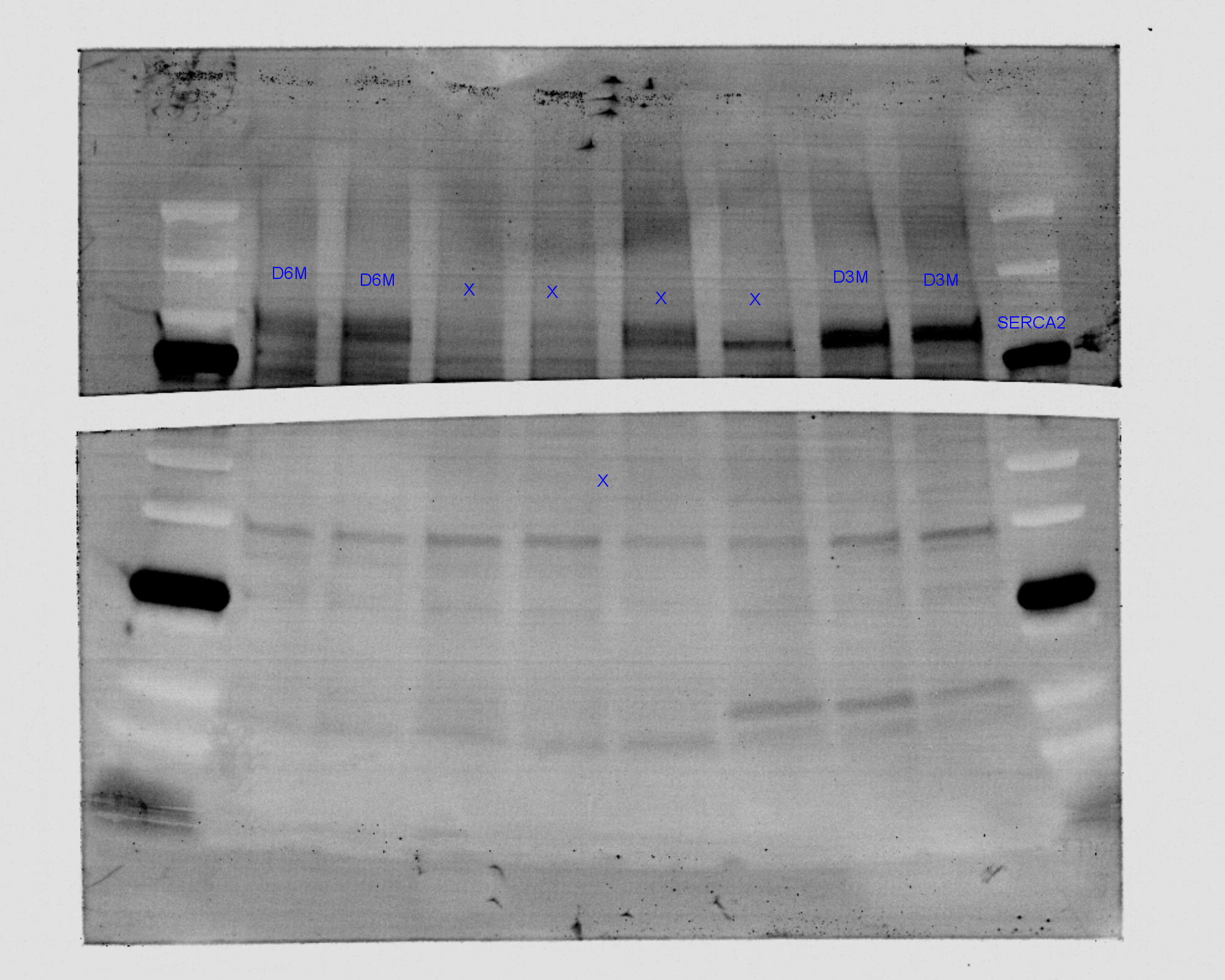

Supplement: Supplementary file 1 [file biomolecules-15-00987-s001.zip › Image_ SERCA2 DM Rats.tif]

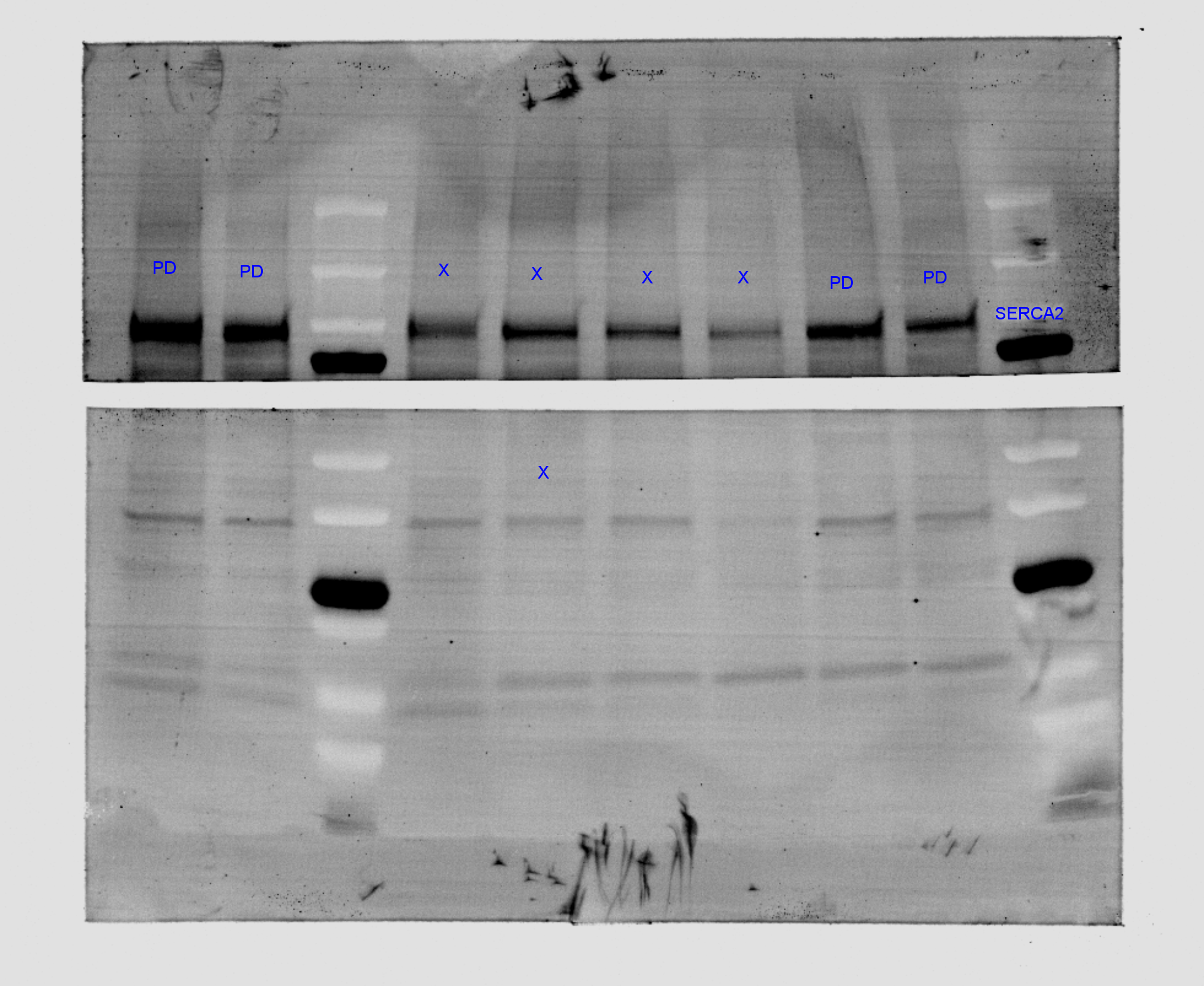

Supplement: Supplementary file 1 [file biomolecules-15-00987-s001.zip › Image_ SERCA2 PD Rats.tif]

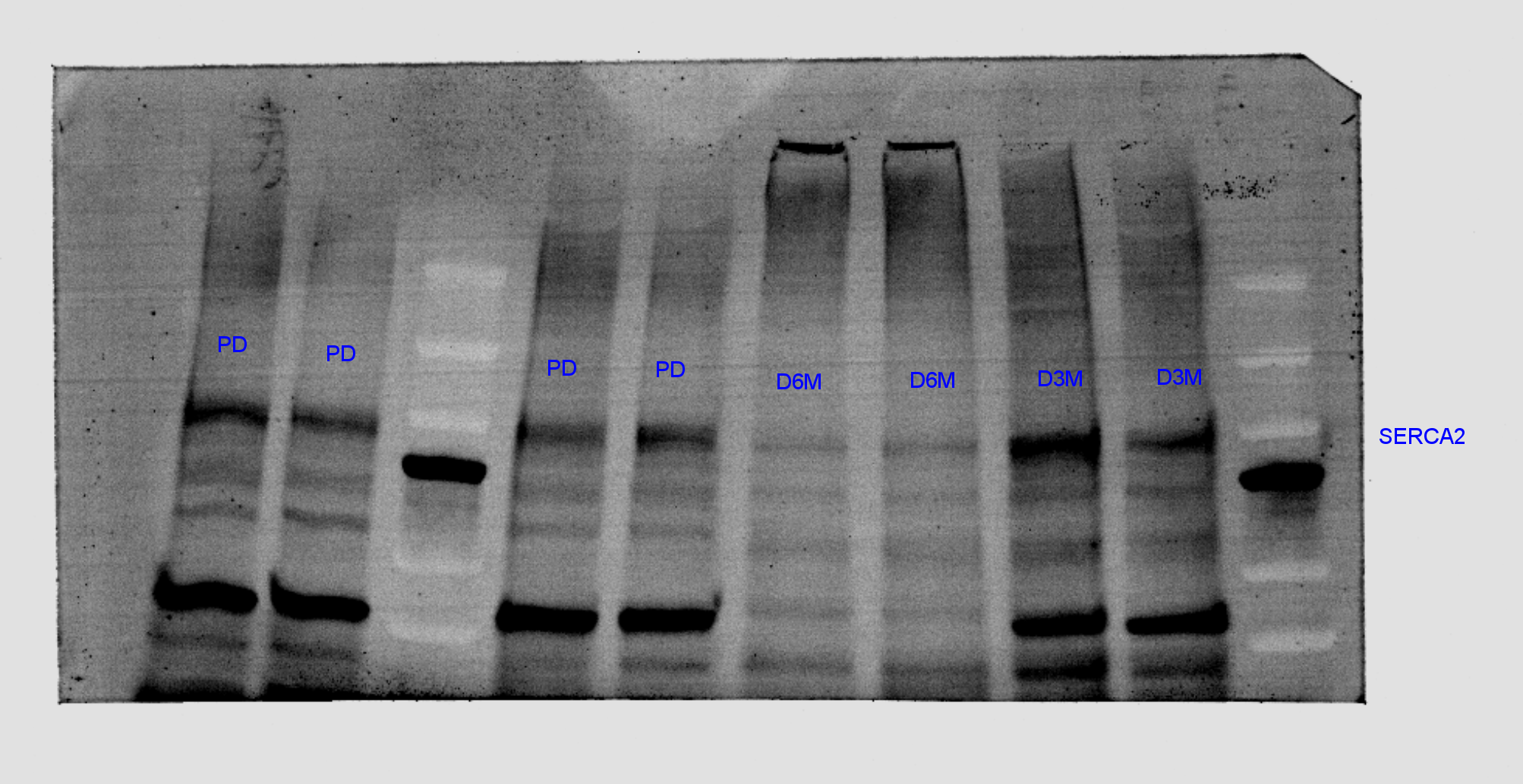

Supplement: Supplementary file 1 [file biomolecules-15-00987-s001.zip › Image_ SERCA2 PHA Activated Lymphocytes PD to DM Rats.tif]

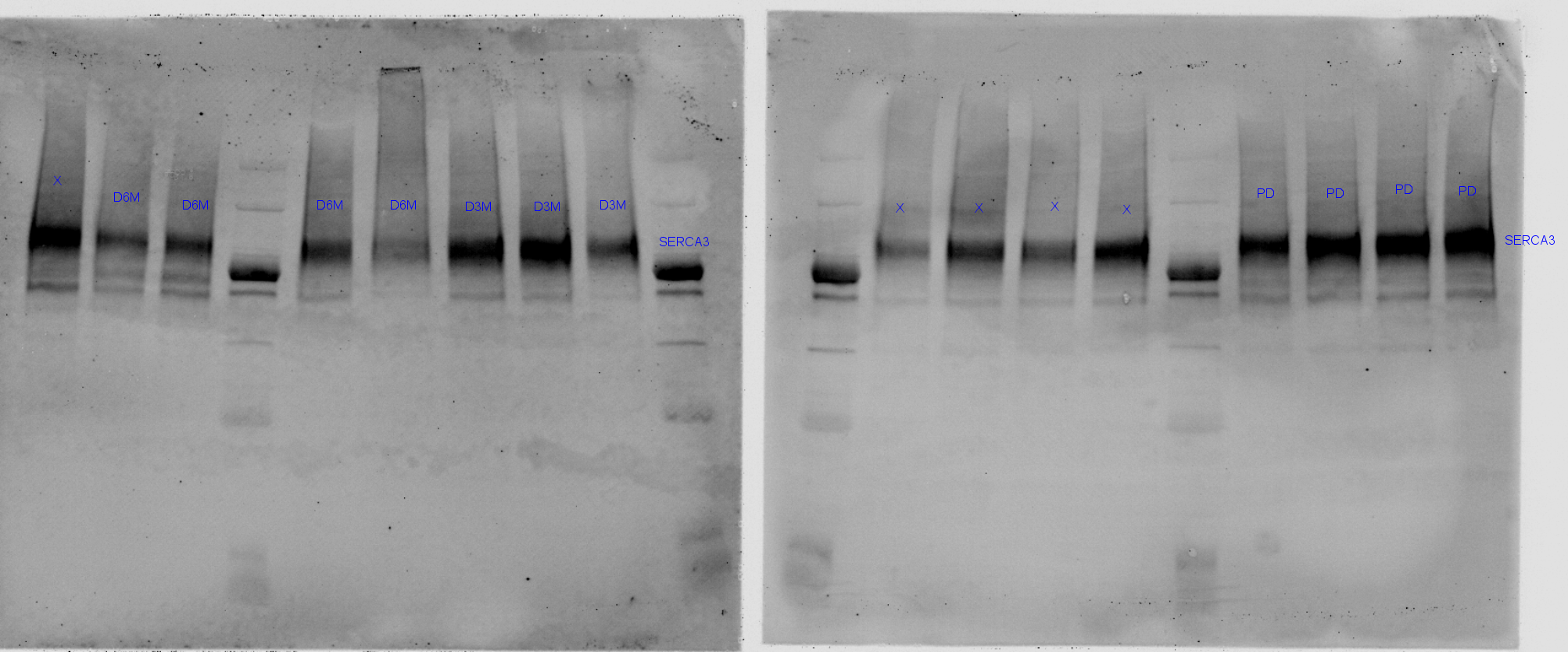

Supplement: Supplementary file 1 [file biomolecules-15-00987-s001.zip › Image_ SERCA3 PD to DM Rats.tif]

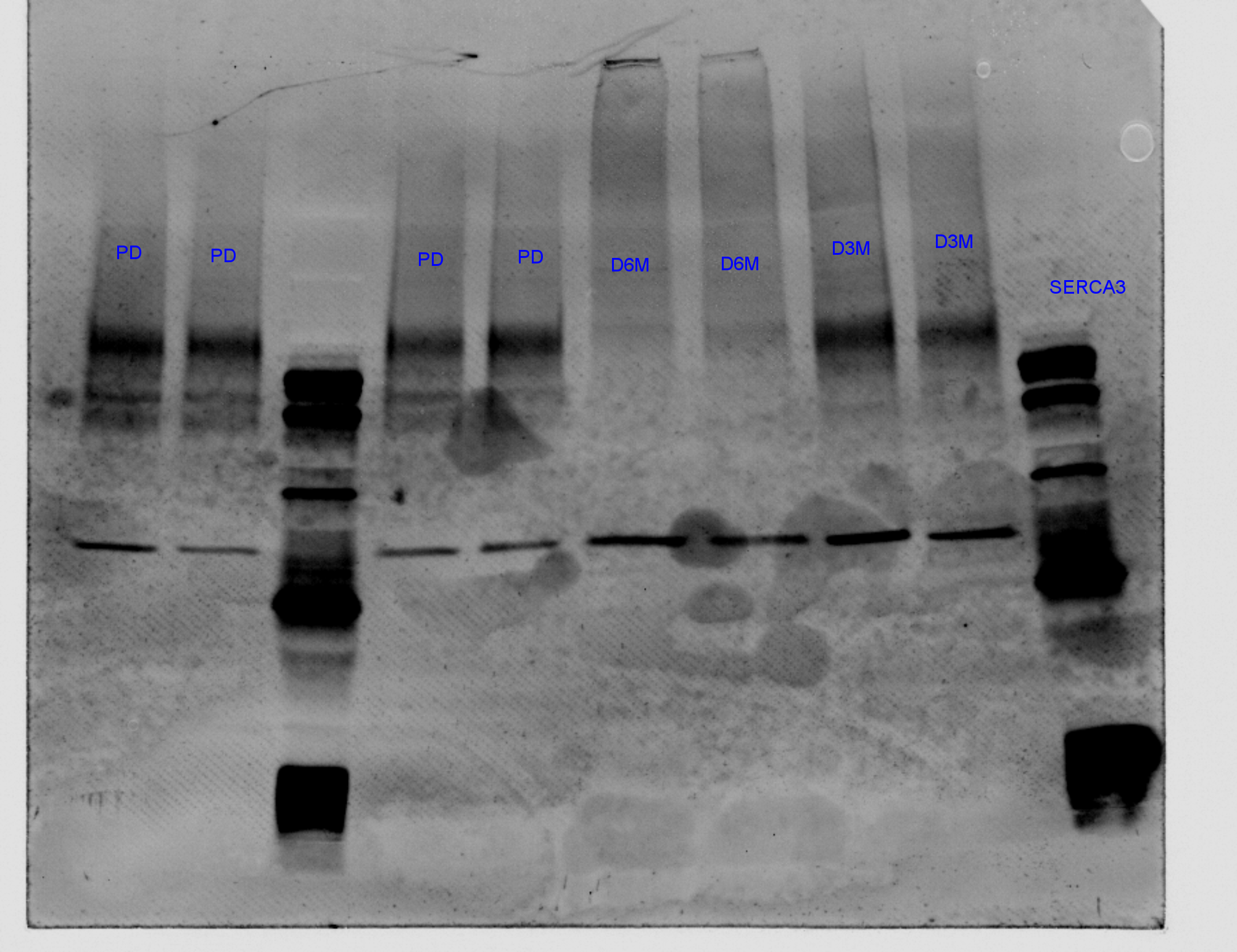

Supplement: Supplementary file 1 [file biomolecules-15-00987-s001.zip › Image_ SERCA3 PHA Activated Lymphocytes PD to DM Rats.tif]

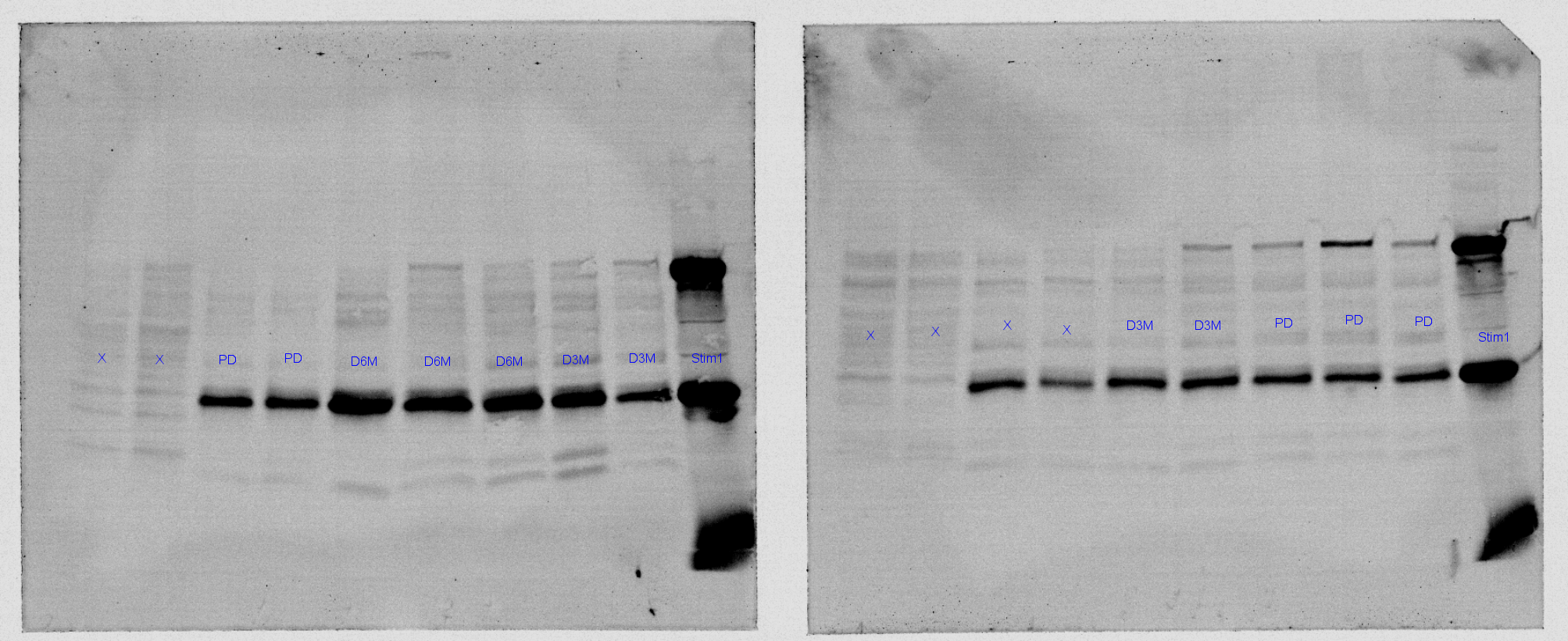

Supplement: Supplementary file 1 [file biomolecules-15-00987-s001.zip › Image_ Stim1 PD to DM Rats.tif]

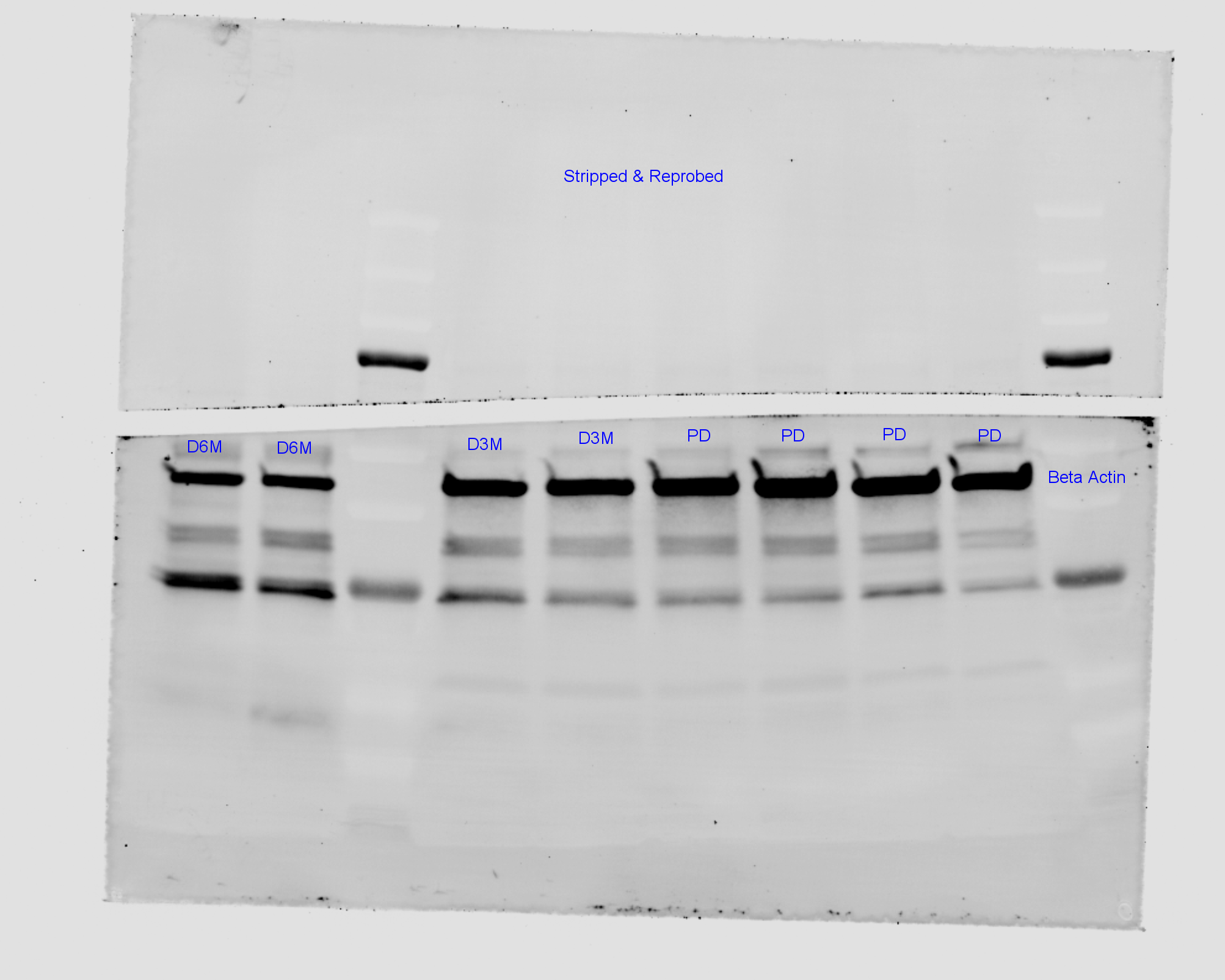

Supplement: Supplementary file 1 [file biomolecules-15-00987-s001.zip › Image_Beta Actin for peIF2 and SERCA2 PD to DM Rats.tif]

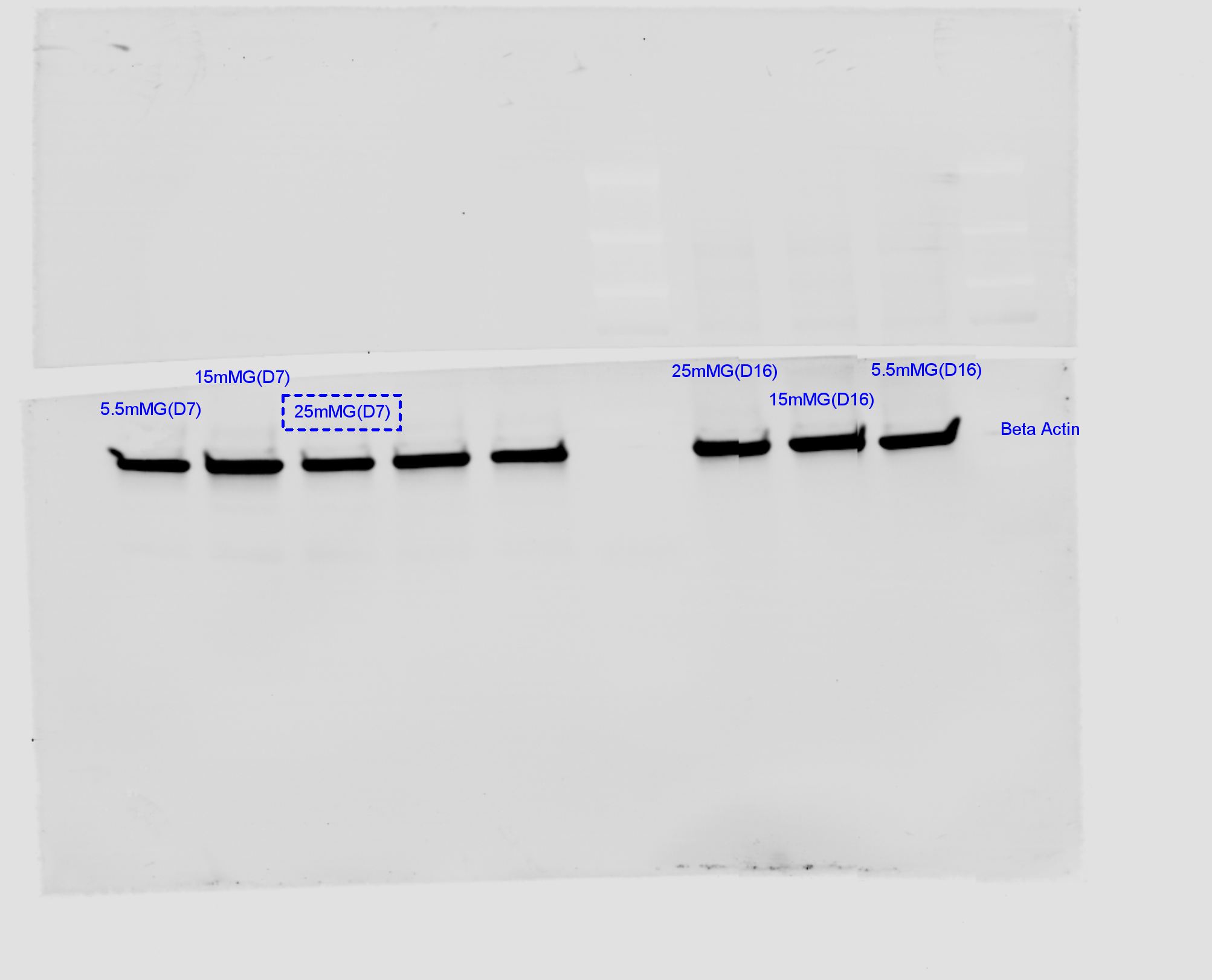

Supplement: Supplementary file 1 [file biomolecules-15-00987-s001.zip › Image_Beta Actin for SERCA2 5.5mM 15mM 25mM Glucose D7 D16.tif]

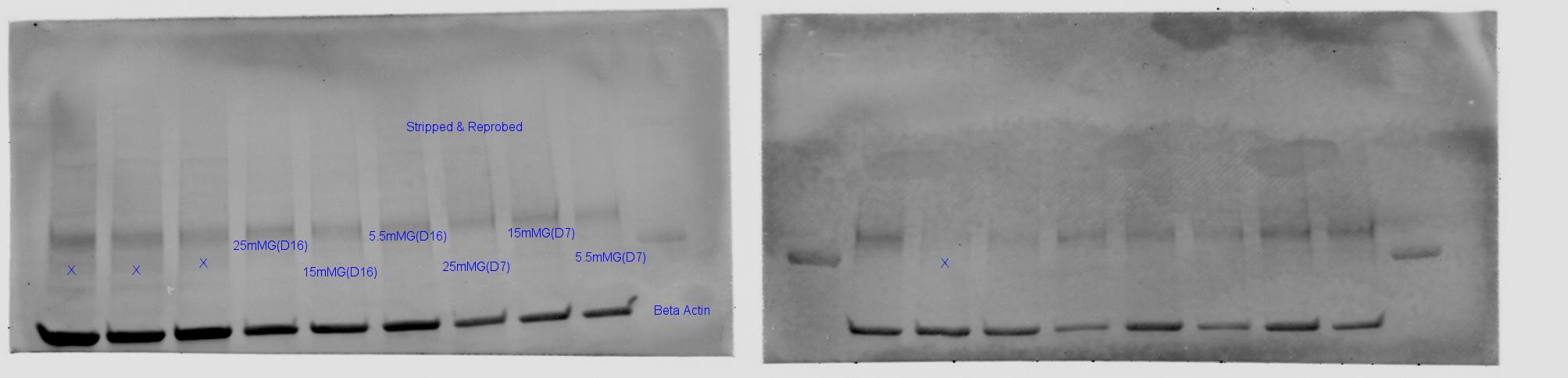

Supplement: Supplementary file 1 [file biomolecules-15-00987-s001.zip › Image_Beta Actin for SERCA3 5.5mM 15mM 25mM Glucose D7 D16.tif]

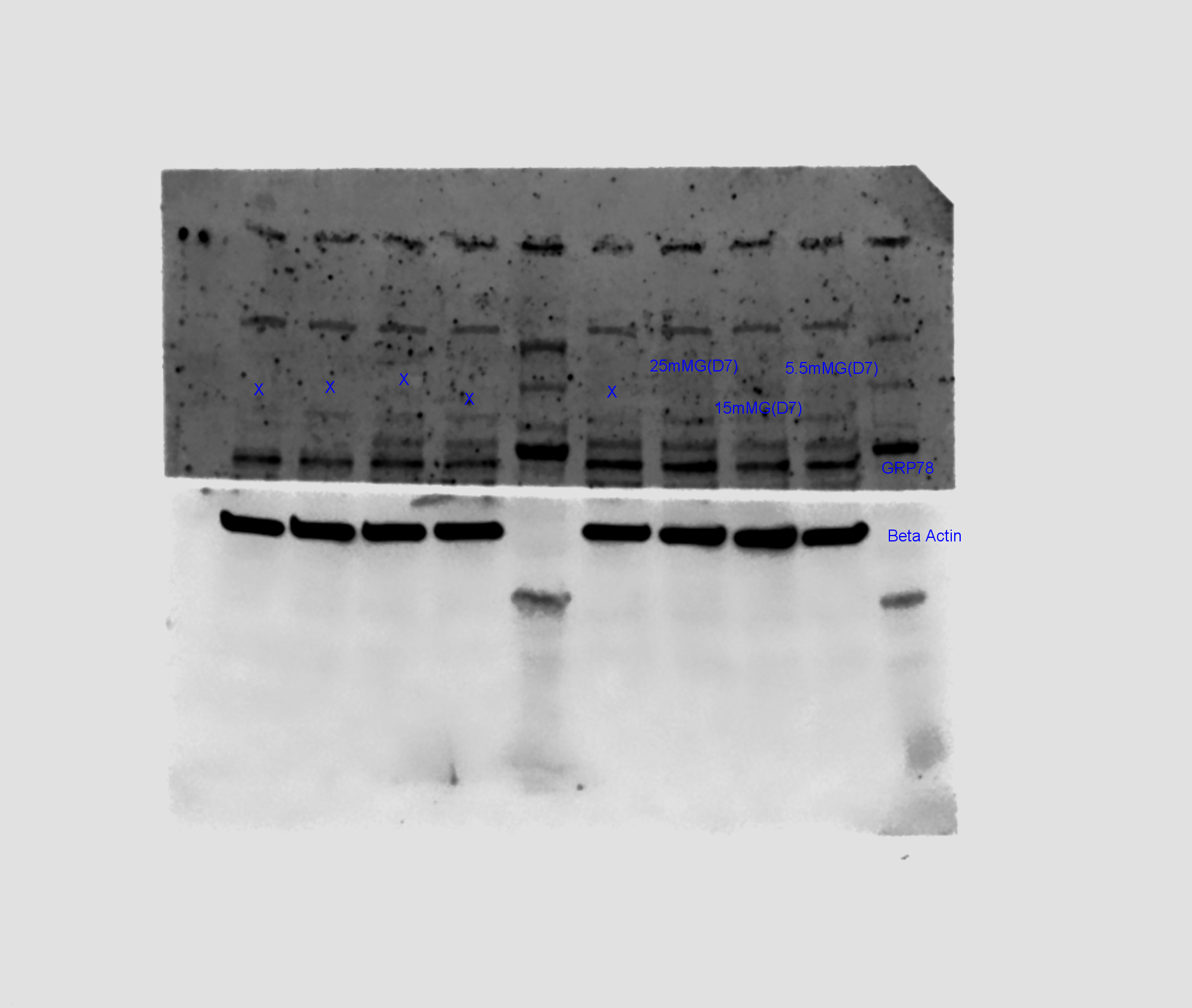

Supplement: Supplementary file 1 [file biomolecules-15-00987-s001.zip › Image_GRP78 and Beta Actin 5.5MM 15mM 25mM Glucose.tif]

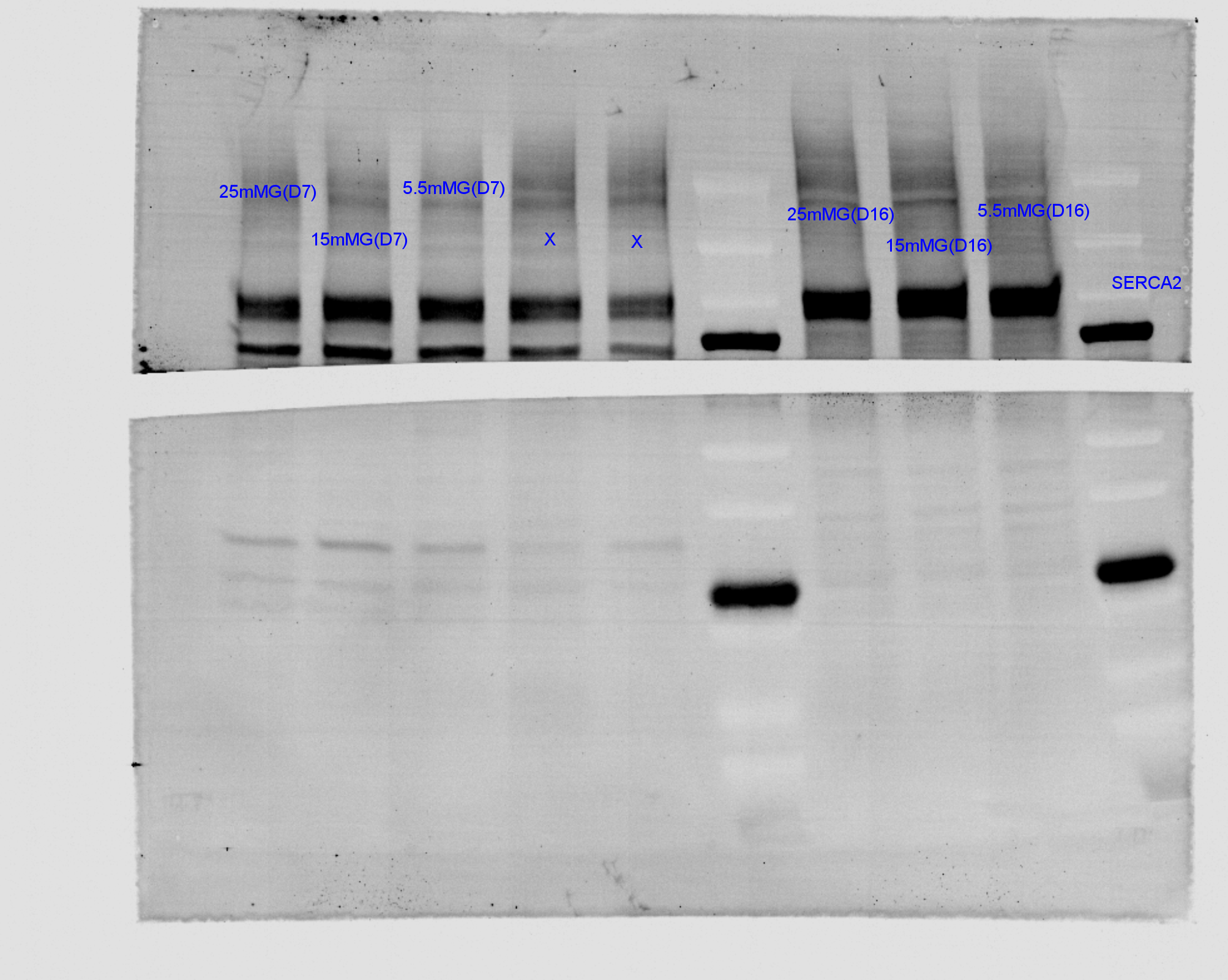

Supplement: Supplementary file 1 [file biomolecules-15-00987-s001.zip › Image_SERCA2 5.5mM 15mM 25mM Glucose D7 D16.tif]

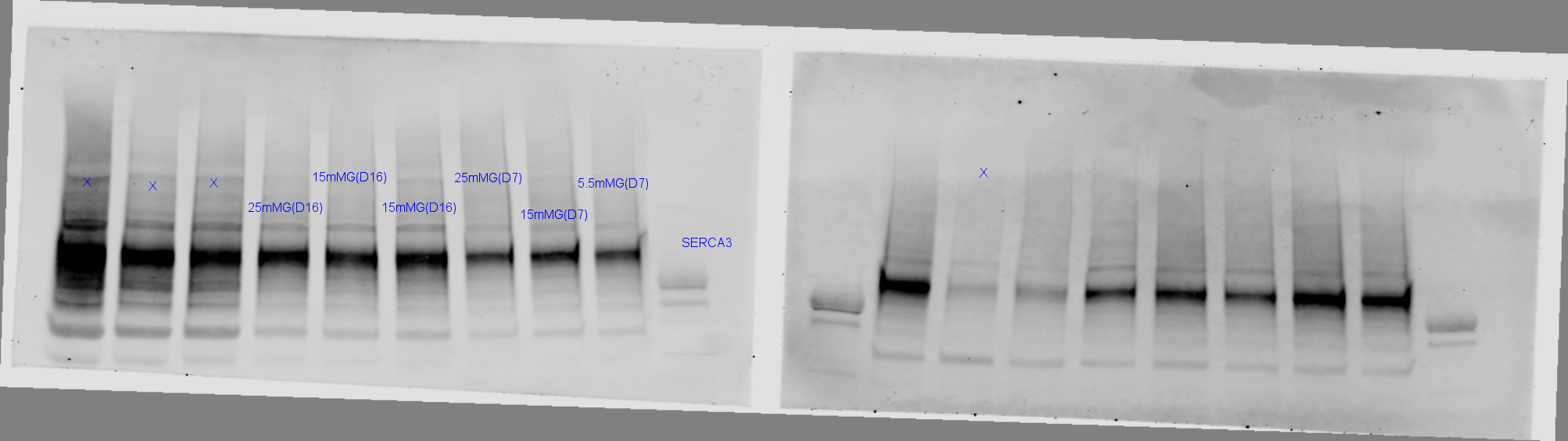

Supplement: Supplementary file 1 [file biomolecules-15-00987-s001.zip › Image_SERCA3 5.5mM 15mM 25mM Glucose D7 D16.tif]
